# Multi-scale datasets for monitoring Mediterranean oak forests from optical remote sensing during the SENTHYMED/MEDOAK experiment in the north of Montpellier (France)

**DOI:** 10.1016/j.dib.2024.110185

**Published:** 2024-02-13

**Authors:** K. Adeline, J.B. Féret, H. Clenet, J.M. Limousin, J.M. Ourcival, F. Mouillot, S. Alleaume, A. Jolivot, X. Briottet, L. Bidel, E. Aria, ATM. Defossez, T. Gaubert, J. Giffard-Carlet, J. Kempf, D. Longepierre, F. Lopez, T. Miraglio, J. Vigouroux, M. Debue

**Affiliations:** a ONERA / DOTA, Université de Toulouse, F-31055 Toulouse, France; b TETIS, Université de Montpellier, AgroParisTech, Cirad, CNRS, INRAE, Montpellier, France; c UMR DYNAFOR, INRAE, Université de Toulouse, 24 Chemin de Borderouge, F-31326 Castanet Tolosan CEDEX, France; d Ecole d'Ingénieurs de Purpan, 75 Voie du TOEC, F-31076 Toulouse, France; e CEFE, Université de Montpellier, CNRS, EPHE, IRD, 34000 Montpellier, France; f UMR IATE, Université de Montpellier, Institut Agro, INRAE, F-34060 Montpellier, France; g CESBIO, Université de Toulouse, CNES/CNRS/INRAE/IRD/UT3-Paul Sabatier, 18, Avenue Edouard Belin, 31401 Toulouse, France

**Keywords:** Oak forests, Species inventory, Canopy plant area index, Leaf traits, Optical properties, UAV-borne LiDAR data, Airborne hyperspectral imagery, Multispectral and hyperspectral satellite data

## Abstract

Mediterranean forests represent critical areas that are increasingly affected by the frequency of droughts and fires, anthropic activities and land use changes. Optical remote sensing data give access to several essential biodiversity variables, such as species traits (related to vegetation biophysical and biochemical composition), which can help to better understand the structure and functioning of these forests. However, their reliability highly depends on the scale of observation and the spectral configuration of the sensor. Thus, the objective of the SENTHYMED/MEDOAK experiment is to provide datasets from leaf to canopy scale in synchronization with remote sensing acquisitions obtained from multi-platform sensors having different spectral characteristics and spatial resolutions. Seven monthly data collections were performed between April and October 2021 (with a complementary one in June 2023) over two forests in the north of Montpellier, France, comprised of two oak endemic species with different phenological dynamics (evergreen: *Quercus ilex* and deciduous: *Quercus pubescens*) and a variability of canopy cover fractions (from dense to open canopy). These collections were coincident with satellite multispectral Sentinel-2 data and one with airborne hyperspectral AVIRIS-Next Generation data. In addition, satellite hyperspectral PRISMA and DESIS were also available for some dates. All these airborne and satellite data are provided from free online download websites. Eight datasets are presented in this paper from thirteen studied forest plots: (1) overstory and understory inventory, (2) 687 canopy plant area index from Li-COR plant canopy analyzers, (3) 1475 *in situ* spectral reflectances (oak canopy, trunk, grass, limestone, etc.) from ASD spectroradiometers, (4) 92 soil moistures and temperatures from IMKO and Campbell probes, (5) 747 leaf-clip optical data from SPAD and DUALEX sensors, (6) 2594 in-lab leaf directional-hemispherical reflectances and transmittances from ASD spectroradiometer coupled with an integrating sphere, (7) 747 in-lab measured leaf water and dry matter content, and additional leaf traits by inversion of the PROSPECT model and (8) UAV-borne LiDAR 3-D point clouds. These datasets can be useful for multi-scale and multi-temporal calibration/validation of high level satellite vegetation products such as species traits, for current and future imaging spectroscopic missions, and by fusing or comparing both multispectral and hyperspectral data. Other targeted applications can be forest 3-D modelling, biodiversity assessment, fire risk prevention and globally vegetation monitoring.

Specifications Table

**Table:** 

Subject	Forestry and Ecology
Specific subject area	*In situ*, laboratory, modelled and multi-platform optical remote sensing data (UAV, airborne and satellite) to monitor Mediterranean oak forests from leaf to canopy scale
Type of data	GIS vectors for the georeferenced locations of the *in situ* datasets (.shp for ESRI shapefile format) Tables for the *in situ*, laboratory and modelled datasets (.txt for text format with tab delimiter) Photos of the *in situ* forest plots and spectroscopic measurements (.jpg for JPEG format) Figures of dataset illustrations/graphs (.png for PNG format) Georeferenced LiDAR 3-D point clouds for UAV-borne data (.laz for LIDAR Data Exchange format)
Data format	Raw and analyzed
Data collection	The SENTHYMED/MEDOAK experiment consisted in seven monthly data collections between April and October 2021, synchronized with Sentinel-2 acquisitions and one AVIRIS-Next Generation airborne campaign, and a complementary ground data collection in June 2023. In addition, punctual PRISMA and DESIS hyperspectral satellite data are available in 2021. Provided datasets include geolocated forest inventories (Trimble and SparkFun RTK Surveyor GPS, Pathfinder office and SW Maps software, QGIS), canopy plant area index (Li-COR LAI-2000/2200, FV2200 software), forest and leaf optical properties (ASD FieldSpec spectroradiometers), soil moisture/temperature (IMKO and Campbell probes), leaf-clip sensor data (Force-A DUALEX and Konica-Minolta SPAD), leaf traits (laboratory facilities, PROSPECT radiative transfer model inversion) and UAV-borne LiDAR 3-D point clouds (Yellowscan Surveyor, DJI Matrice 600 Pro UAV).
Data source location	For the in-situ data collection and UAV-borne acquisitions: • Localization: Puéchabon (CNRS experimental site in national forest) and Pic Saint-Loup (national forest) • Region, Country: Occitanie, France • Latitude and longitude: Puéchabon (Long. 3.5866° E, Lat. 43.7342° N) and Pic Saint-Loup (Long. 3.7961° E, Lat. 43.7751° N) For the airborne and satellite remote sensing data (all processed in Level 2, atmospherically corrected at-surface reflectance products): • The AVIRIS-Next Generation data can be downloaded from https://ares-observatory.ch/esa_chime_mission_2021/ (image rasters, ENVI format) • PRISMA and DESIS data can be downloaded respectively from PRISMA portal at https://www.asi.it/en/earth-science/prisma/ (image rasters, .he5 for Hierarchical Data Format, Release 5) and EOWEB portal at https://eoweb.dlr.de/egp/ (image rasters, .tif for GeoTIFF format) • Sentinel-2 data can be downloaded from the THEIA portal at https://www.theia-land.fr/en/product/sentinel-2-surface-reflectance (image rasters, .tif for GeoTIFF format)
Data accessibility	Repository name: SEDOO
	Data identification number: • Forest inventory: https://doi.org/10.6096/8005 • Canopy plant area index: https://doi.org/10.6096/8007 • Forest optical properties : https://doi.org/10.6096/8006 • Soil moisture : https://doi.org/10.6096/8001 • Leaf-clip optical sensor data : https://doi.org/10.6096/8002 • Leaf optical properties: https://doi.org/10.6096/8004 • Leaf traits : https://doi.org/10.6096/8003 • UAV-borne LiDAR 3-D point clouds : https://doi.org/10.15454/AGBW7G, https://doi.org/10.15454/DMYWPB Direct URL to all data: https://remotetree.sedoo.fr/catalogue/ Instructions for accessing the datasets on the website: the datasets are visible under the search menu through projects and then by selecting FOREST/SENTHYMED

## 1 Value of the Data

• These datasets were collected to provide calibration/validation data for methods aiming at linking ground observations on Mediterranean forests with multi-scale, multi-temporal and multi-platform remote sensing data.
• These datasets are beneficial for researchers in vegetation remote sensing, modelling and ecophysiology communities. They are also of interest for space agencies designing new missions and requiring ground truth data for calibration/validation of high-level vegetation products conception.
• These datasets can be reused for many applications and algorithmic developments dedicated to forest fire risk, biodiversity assessment, forest 3D modelling, species classification and species trait estimation, multi-sensor data fusion, scaling up in vegetation studies and vegetation monitoring.

## 2 Background

The objective of these datasets was to gather relevant *in situ* data as part of the SENTHYMED/MEDOAK experiments. These datasets are intended for the validation of vegetation products derived from upcoming imaging spectroscopy missions. SENTHYMED [1] aims at studying the “complementarity between Sentinel-2 multi-temporal imagery and hyperspectral imagers for a better monitoring of the functional traits of Mediterranean forests”, in support to the CNES BIODIVERSITY mission [2]. MEDOAK was organized as part of the airborne and ground measurement campaign in support of the Copernicus High Priority Candidate Mission – CHIME (Copernicus Hyperspectral Imaging Mission for the Environment) from ESA [3] and NASA Surface Biology and Geology (SBG) mission [4]. MEDOAK aims at measuring species traits to assess biodiversity-ecosystem functioning from spectroscopic imagery, and provides a study case in the French MEDiterranean basin with OAK forests. Targeted vegetation products are the mapping and monitoring of species traits (or essential biodiversity variables [5]) for Mediterranean oaks at leaf and canopy scale. These datasets will provide a better understanding of Mediterranean forest conditions from a multi-scale (*in situ*, UAV, airborne, satellite) and multi-temporal approach with the possibility to accurately account for the optical and structural properties found in this ecosystem type for modelling purposes.

## 3 Data Description

Eight datasets are described in Sections 3.1 to 3.8. Table 1 provides a general naming nomenclature of several acronyms (highlighted in capital letters) that will be used throughout the paper to harmonize the annotations among the different data types. An identification tag (here ID_LOC) is used to link georeferenced vector files (shapefiles) with non-georeferenced files (text files). All georeferenced *in situ* data were defined with the coordinate reference system WGS1984 UTM 31N (EPSG: 32631) while UAV-borne LiDAR used the RGF93/Lambert 93 coordinate reference system. The two study sites Puéchabon and Pic Saint Loup are hereafter referred to as PUE and PSL. In the datasets, if “NA” is indicated, it means that no value or information is applicable.

**Table 1:** General naming nomenclature.

Name	Value
SAMPLING_DATE	Date of leaf sampling: YYYYMMDD (YYYY: year, MM: month, DD: day)
SAMPLING_TIME	Time of leaf sampling: HH:MM:SS (HH: hour, MM: minute, SS: second, local time)
MEAS_DATE	Date of measurement: YYYYMMDD (YYYY: year, MM: month, DD: day)
MEAS_TIME	Time of measurement: HH:MM:SS (HH: hour, MM: minute, SS: second, local time)
SITE	Acronym of the site: PUE or PSL
SITE_PLOT	Identification of the plot. For PSL: number (ex: 11); for PUE: ICOS Ecosystem station FR-Pue CP1 or CP2
SURF_TYPE	Category of the measured surface: LEAF, TRUNK, CANOPY (above canopy), GRASS, LIMESTONE, DIRTROAD, BARESOIL, LITTER (leaf litter), MOSS, MIXGL (mix of grass and limestone), MIXGLI (mix of grass and litter), MIXBL (mix of bare soil and limestone), MIXBLI (mix of grass and litter), MIXBG (mix of bare soil and grass), MIXLIL (mix of litter and limestone), MIXGLB (mix of grass, limestone and bare soil), MIXGLIL (mix of grass, litter and limestone)
SPECIES	Identification of the vegetation species: QP (Quercus pubescens), QI (Quercus ilex), JU (Juniperus oxycedrus), PI (Pistacia terebinthus), BU (Buxus sempervirens), PH (Phillyrea latifolia), TH (Thyme), RO (Rosemary), BR (Bramble)
TREE	Identification of the tree on the plot. For PSL: D or M + number (D stands for dominant tree and M for non dominant tree, ex: D1); for PUE: number (based on ICOS inventory, ex: 290)
TREE_LEAF	Number of the leaf for a tree: L + number (from 1 to 5, ex: L1, L2, etc.)
TREE_LEAF_AGE	Global age of the leaf: Y (young leaf from 2021) or O (old leaf from previous years)
PAI_DEVICE_LAB	Owner of the PAI measurement device: CEFE, DYNAFOR or CESBIO
PAI_DEVICE_TYPE	LICOR_LAI2000 or LICOR_LAI2200
PAI_MEAS_TYPE	Type of PAI measurement: ABOVE (above canopy measurements) or BELOW (below canopy measurements)
SPECTRAL_DEVICE_LAB	Owner of the spectroradiometer instrument: ONERA, DYNAFOR or TETIS
SPECTRAL_DEVICE_TYPE	ASD_FIELDSPEC3 or ASD_FIELDSPEC4HR
SPECTRAL_DEVICE_ACCESSORY	Type of the accessory used or not for the spectroscopic measurements: BARE (bare fiber), OPTIC + number (ex: OPTIC8 for 8-degree fore optic field of view), CONTACT (contact probe), CLIP (leaf clip), SPHERE (integrating sphere)
SPECTRAL_ACQUI_TYPE	Type of spectroscopic acquisition: P (point), A (area) or T (transect)
SPECTRAL_MEAS_TYPE	Type of spectroscopic measurement: R (reflectance) or T (transmittance)
MOISTURE_DEVICE_LAB	Owner of the soil moisture probe: ONERA or CEFE
MOISTURE_DEVICE_TYPE	IMKOHD2_TDR or CAMPBELL_CS616
LEAFCLIP_DEVICE_LAB	Owner of the leaf-clip device: CEFE, TETIS or CEFE&TETIS
LEAFCLIP_DEVICE_TYPE	KONICAMINOLTA_SPAD, FORCEA_DUALEX or KONICAMINOLTA_SPAD&FORCEA_DUALEX
LEAFTRAIT_LAB	Owner of the measurement instruments used to determine leaf traits: TETIS
ID_MEAS	Number of the measurement. For PAI: between 1 and 25 for BELOW and 0 for ABOVE; for SPECTRAL: number of the acquisition; for soil moisture: between 1 and 2; for understory inventory: position between 1 and 25
ID_LOC	Unique identification tag for each location of PAI, forest optical properties and soil moisture measurements, and also the location where the leaves were sampled
XCOORD	X geographic coordinate of the position (units: meters)
YCOORD	Y geographic coordinates of the position (units: meters)
COMMENTS	Comments on the acquired data

### 3.1 Forest inventory

A zip folder named “SENTHYMED_MEDOAK_forest_inventory.zip” contains ten files:

• SENTHYMED_MEDOAK_forest_plot_coord_P.shp (data type: positions)
• SENTHYMED_MEDOAK_forest_plot_coord_A.shp (data type: areas)
• SENTHYMED_MEDOAK_inventory_overstory_coord_P.shp (data type: positions)
• SENTHYMED_MEDOAK_inventory_overstory_coord_A.shp (data type: areas)
• SENTHYMED_MEDOAK_inventory_understory_coord_P.shp (data type: positions)

The two first files include the forest plot locations (i.e. centers and circular areas) while the three others include the forest inventory for the overstory (tree positions and crown area delineations) and understory (ground types and/or vegetation species). The attribute table includes SITE, SITE_PLOT, XCOORD and YCOORD for the first file, SITE and SITE_PLOT for the second, SITE, SITE_PLOT, SPECIES, XCOORD and YCOORD for the third, SITE, SITE_PLOT, SPECIES and COMMENTS for the fourth, and finally SITE, SITE_PLOT, SURF_TYPE, SPECIES, ID_MEAS, XCOORD and YCOORD for the fifth (cf. Table 1 for the used acronyms).

• SENTHYMED_MEDOAK_forest_plot_photos.zip

This zipfile includes several folders with photos (JPEG format) taken in the field of the forest plots. The name of each photo contains at least MEAS_DATE, MEAS_TIME, SITE, SITE_PLOT and sometimes TREE (cf. Table 1 for the used acronyms).

**Fig. 1:**
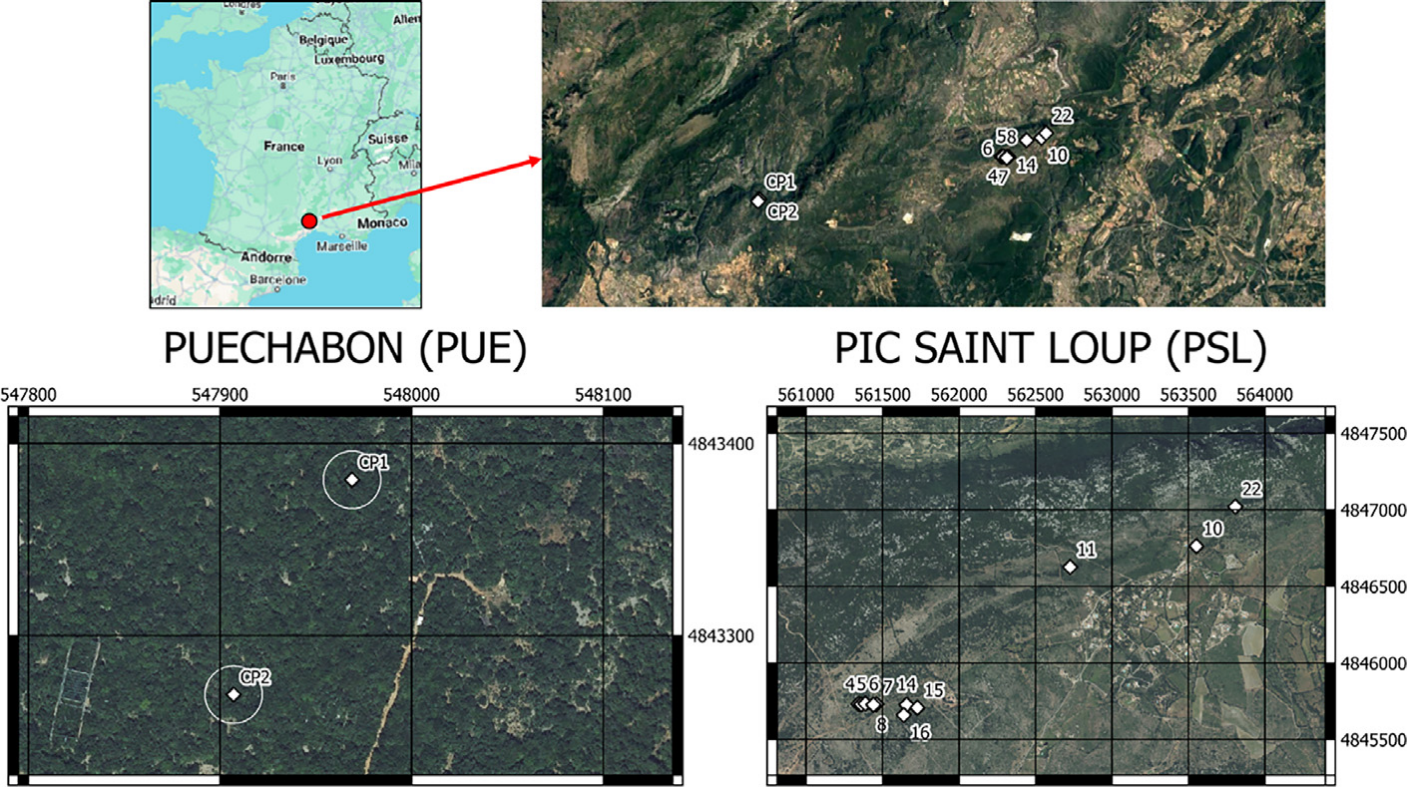
Location of the two forest sites and the plots for each site (used backgrounds: from Google for the top row and from IGN BD ORTHO® at 20 cm spatial resolution for the bottom row).
• SENTHYMED_MEDOAK_forest_inventory_figure2.png (cf. Fig. 2)

• SENTHYMED_MEDOAK_forest_inventory_figure1.png (cf. Fig. 1)

**Fig. 2:**
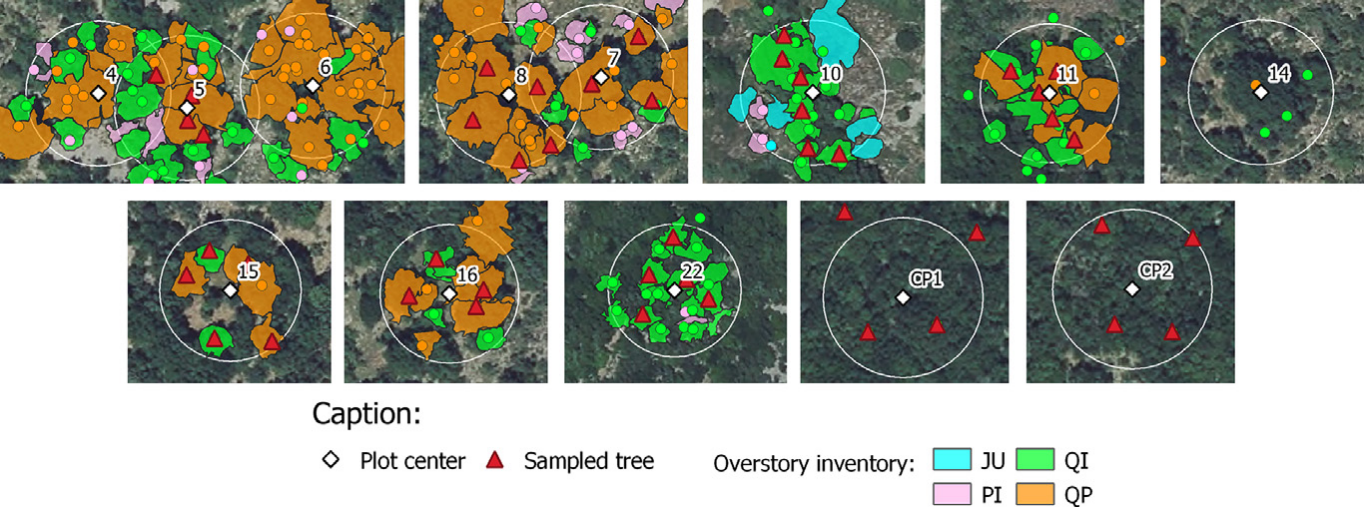
Forest overstory inventory data (tree positions and tree crown delineated areas, cf. Table 1 for used species acronyms) and location of the trees where were sampled the leaves (used background: IGN BD ORTHO® at 20 cm spatial resolution).
• SENTHYMED_MEDOAK_forest_inventory_figure3.png (cf. Fig. 3)

**Fig. 3:**
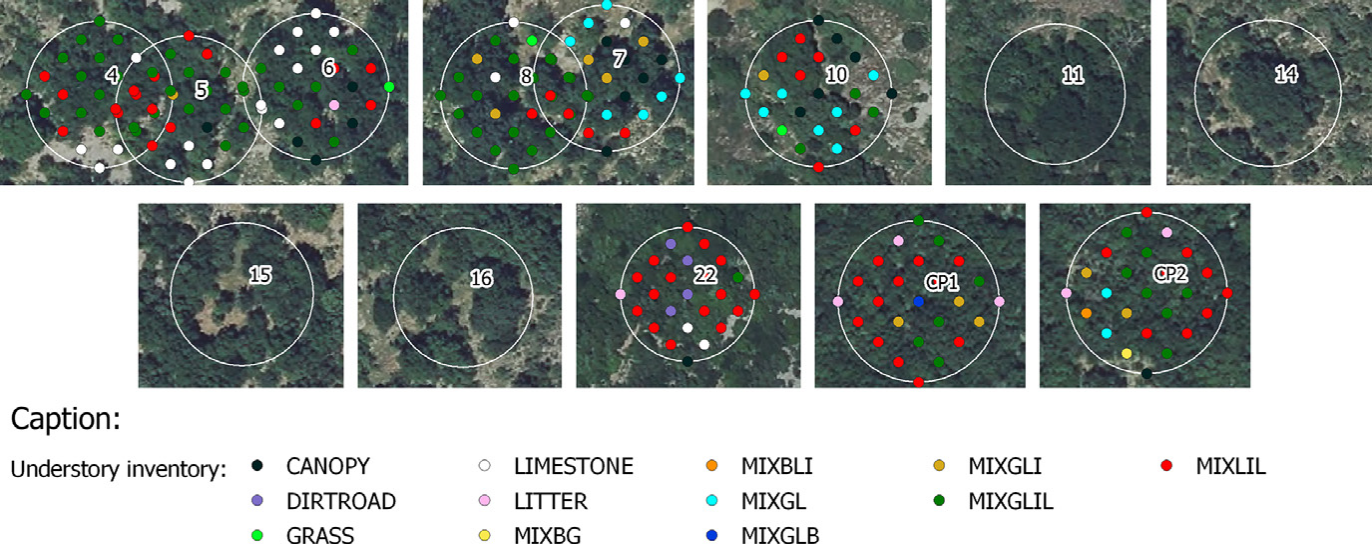
Forest understory inventory data (cf. Table 1 for used surface type acronyms, used background: IGN BD ORTHO® at 20 cm spatial resolution).
• SENTHYMED_MEDOAK_forest_inventory_figure4.png (cf. Fig. 4)

**Fig. 4:**
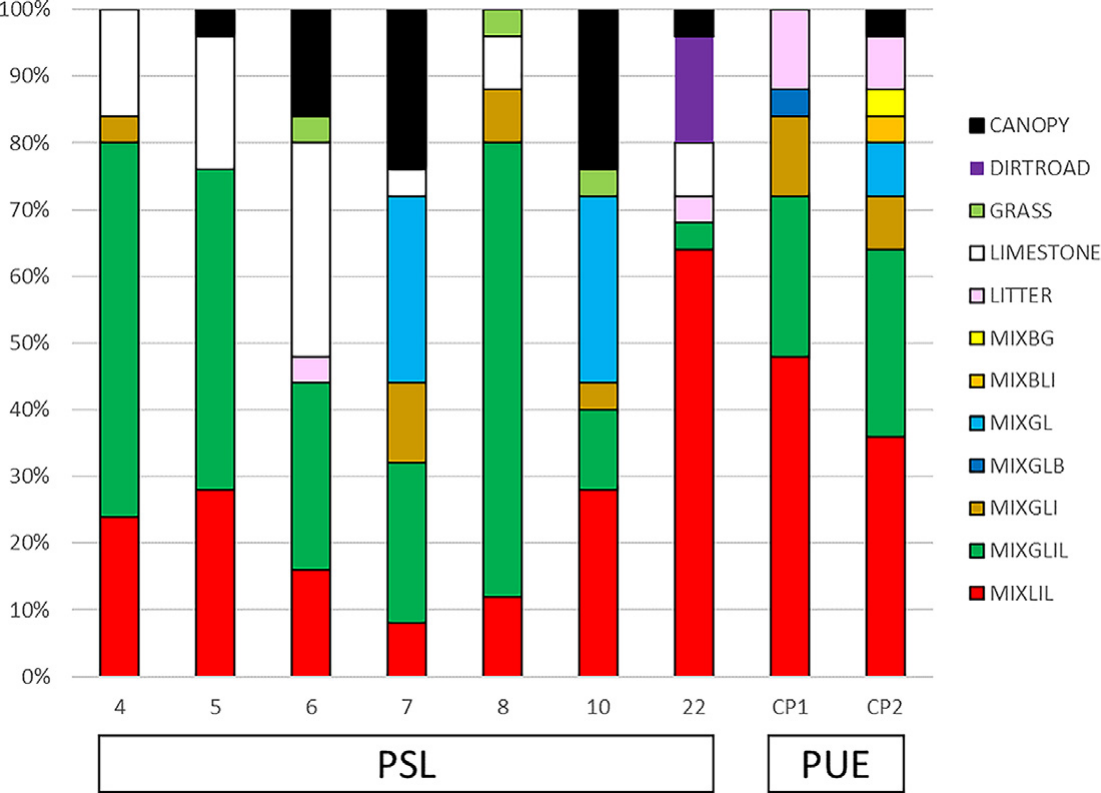
Statistics of the forest understory inventory for each visited plot in June 2023 (cf. Table 1 for used surface type acronyms).

### 3.2 Canopy plant area index

The zipped folder “SENTHYMED_MEDOAK_canopy_plant_area_index.zip” contains three files:

• SENTHYMED_MEDOAK_canopy_plant_area_index_data.txt

This file gives the post-processed PAI values and its table columns give information on ID_LOC, MEAS_DATE, MEAS_TIME, SITE, SITE_PLOT, PAI_DEVICE_LAB, PAI_DEVICE_TYPE, PAI_MEAS_TYPE, ID_MEAS, PAI (effective PAI, units: m^2^.m^−2^), GAP1, GAP2, GAP3, GAP4 and GAP5 (five gap fraction values), BAD_READINGS (“NA” meaning non applicable and “ERROR” error in the measurement). The ID_LOC naming is as follows: SITE + “_” + SITE_PLOT + “_MEAS”+ ID_MEAS (ex: PSL_4_MEAS1). Please refer to Table 1 for the used acronyms.

• SENTHYMED_MEDOAK_canopy_plant_area_index_coord_P.shp (data type: positions)

This file provides the locations where PAI measurements were performed with attribute table columns giving information on ID_LOC, PAI_MEAS_TYPE (truncated to PAI_MEAS_T), XCOORD and YCOORD. Note that ID_MEAS equalling zero corresponds to PAI_MEAS_TYPE equalling ABOVE. Sometimes, the same location was chosen for several plots given their spatial proximity (ex: PSL_4-5-6-7-8-14-15-16_MEAS0 and PUE_CP1-CP2_MEAS0). Please refer to Table 1 for the used acronyms.

**Fig. 5:**
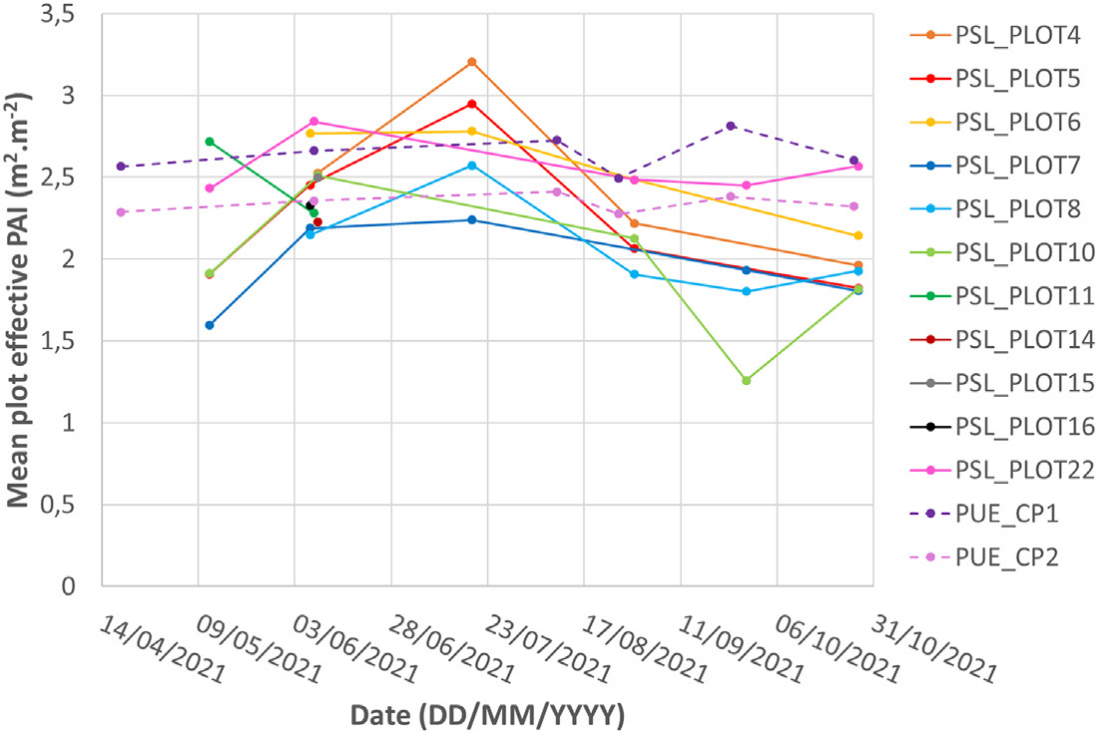
Phenological variations over the year 2021 of the mean plot PAI values (excluding bad readings and repetitions) for the two sites.

• SENTHYMED_MEDOAK_canopy_plant_area_index_figure1.png (cf. Fig. 5)

### 3.3 Forest optical properties

The zipped folder “SENTHYMED_MEDOAK_forest_optical_properties.zip” contains seven files:

• SENTHYMED_MEDOAK_forest_optical_properties_data.txt

This file corresponds to a table including *in situ* measured optical properties along with information on ID_LOC, MEAS_DATE, MEAS_TIME, SPECTRAL_DEVICE_LAB, SPECTRAL_DEVICE_TYPE, SPECTRAL_DEVICE_ACCESSORY, SPECTRAL_ACQUI_TYPE, SPECTRAL_MEAS_TYPE, SITE, SURF_TYPE, SPECIES and ID_MEAS, then each column represents the reflectance value for spectral bands from 350 nm to 2500 nm with a spectral step of 1nm. The naming of ID_LOC does not follow a particular rule but generally includes MEAS_DATE, SITE and SURF_TYPE. Actually, it depends on the GPS acquisition type (P, A or T) or if the GPS position is unknown (ID_LOC = NA). In some cases, “SEVERAL” is used to mention that several measurements were taken in the same area. Also, a numbering can be attributed to the SURF_TYPE for transect acquisitions acquired several times on the same material (ex: GRASS1 and GRASS2). “PUE_CANOPY_PLATFORM” name was used to mention measurements at several dates on the same location. More information can be found in Tables 2 and 1 for the used acronyms.

**Table 2:** Summary of the *in situ* measured optical properties with their associated photos and geographic locations when available. Refer to Table 1 for the used acronyms.

MEAS_DATE SITE	COMMENTS
20210402 PUE	Measurements performed above the QI canopy on a scaffolding platform, large ONERA spectralon for calibration, one GPS point (PUE_CANOPY_QI_PLATFORM), three photos
20210402 PSL	Measurements to characterize the understory close to PLOT 22 (CANOPY for JU, TH, BU and QI, GRASS, DIRTROAD, MIXGL, LIMESTONE), small DYNAFOR spectralon for calibration, one GPS area encompassing the area where the punctual measurements were performed (no precise location, 20210402_PSL_SEVERAL), no photo
20210512 PUE	Measurements performed above the QI canopy on a scaffolding platform (same location as the previous April measurement, PUE_CANOPY_QI_PLATFORM), small DYNAFOR spectralon for calibration, one GPS point (equivalent to April), three photos
	Measurements on a QI trunk performed close to the dirty road in a dense canopy area, small DYNAFOR spectralon for calibration, one GPS point (20210512_PUE_TRUNK_QI), three photos
20210608 PUE	Measurements performed above the QI canopy on a scaffolding platform (same location as the previous April/May measurements, PUE_CANOPY_QI_PLATFORM), small DYNAFOR spectralon for calibration, one GPS point (equivalent to April/May), four photos
	Measurements performed on the dirty road and in an open instrumented area (CANOPY for RO, DIRTROAD, GRASS, LITTER, MIXBG, MIXBL, MIXGL, MIXGLB), small DYNAFOR spectralon for calibration, one GPS area encompassing the area where the punctual measurements were performed (no precise location, 20210608_PUE_SEVERAL), four photos
	Measurements performed on the understory (LIMESTONE, MIXLIL), no GPS location, one photo
	Measurement on a QI trunk performed on CP1, small DYNAFOR spectralon for calibration, no GPS location, two photos
20210719 PSL	Measurements to characterize the understory performed close to PLOT 10 (JU & QI CANOPY, LIMESTONE, GRASS), large ONERA spectralon for calibration, four GPS points (20210719_PSL_CANOPY_JU, 20210719_PSL_CANOPY_QI1, 20210719_PSL_CANOPY_QI2, 20210719_PSL_LIMESTONE) and two GPS transects (20210719_PSL_GRASS1, 20210719_PSL_GRASS2), no photo
20210720 PSL	Measurements to characterize the understory performed close to PLOT 7 (JU & PI CANOPY, LIMESTONE, MIXBL, DIRTROAD, MIXGL), large ONERA spectralon for calibration, four GPS points (20210720_PSL_CANOPY_JU, 20210720_PSL_CANOPY_PI, 20210720_PSL_LIMESTONE, 20210720_PSL_MIXBL) and three GPS transects (20210720_PSL_DIRTROAD, 20210720_PSL_MIXGL1, 20210720_PSL_MIXGL2), seven photos
20210830 PSL	Measurements to characterize the understory performed close to PLOT 10 (JU CANOPY, GRASS), large ONERA spectralon for calibration, two GPS points (20210830_PSL_CANOPY_JU1, 20210830_PSL_CANOPY_JU2) and two GPS transects (20210830_PSL_GRASS1, 20210830_PSL_GRASS2), no photo
20210831 PSL	Measurements to characterize the understory performed close to a dirt road close to the area with PLOT 1-8 (JU & PI CANOPY, DIRTROAD, LIMESTONE, MIXGLB, MIXBL), large ONERA spectralon for calibration, four GPS points (20210831_PSL_CANOPY_JU, 20210831_PSL_CANOPY_PI1, 20210831_PSL_CANOPY_PI2, 20210831_PSL_MIXBL) and four GPS transects (20210831_PSL_MIXGLB1, 20210831_PSL_ MIXGLB2, 20210831_PSL_LIMESTONE, 20210831_PSL_DIRTROAD), no photo
20230607 PSL	Measurements performed in plot 7-8 (QP TRUNK, LIMESTONE, LITTER, MOSS), no GPS location, no photo

• SENTHYMED_MEDOAK_forest_optical_properties_coord_P.shp (data type: positions)
• SENTHYMED_MEDOAK_forest_optical_properties_coord_A.shp (data type: areas)
• SENTHYMED_MEDOAK_forest_optical_properties_coord_T.shp (data type: transects)

These three files allow geolocating all in situ measurements performed to characterize canopy, understory, ground and surroundings of the plots. Each attribute table includes information on ID_LOC. XCOORD and YCOORD are only provided for the GPS positions. Please refer to Table 1 for the used acronyms.

• SENTHYMED_MEDOAK_forest_optical_properties_photos.zip

This zipped folder contains photos of the measured surface when available. File naming contains at least MEAS_DATE, SITE and SURF_TYPE. Please refer to Table 2 for more information and Table 1 for the used acronyms.

**Fig. 6:**
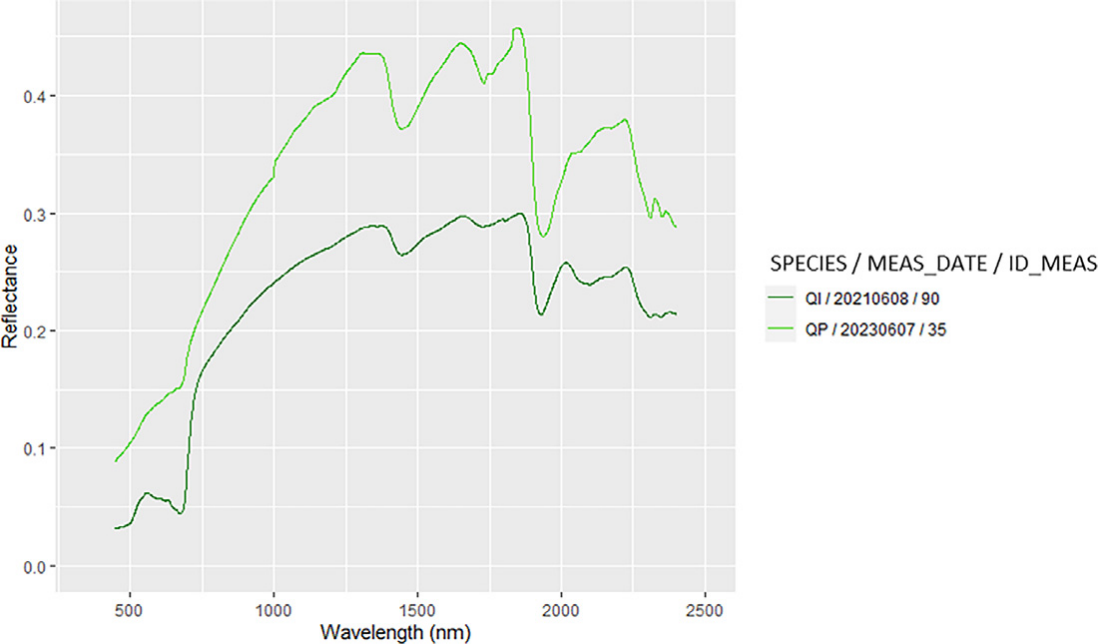
Examples of measured spectra of QI and QP trunk in June 2023 (cf. Table 1 for used acronyms).
• SENTHYMED_MEDOAK_forest_optical_properties_figure2.png (cf. Fig. 7)

• SENTHYMED_MEDOAK_forest_optical_properties_figure1.png (cf. Fig. 6)

**Fig. 7:**
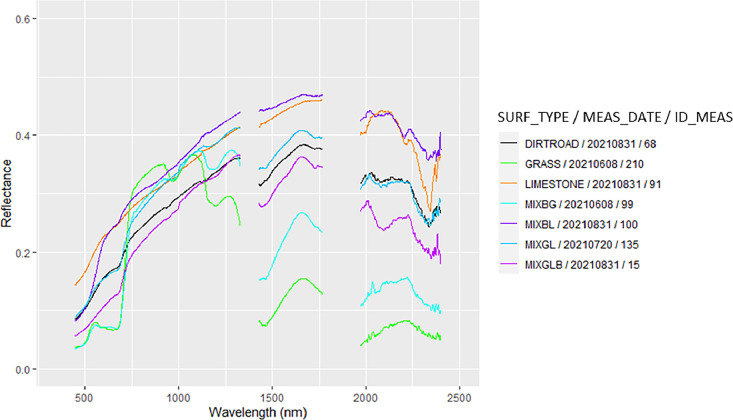
Examples of measured spectra for different understory types at different dates (cf. Table 1 for used acronyms).

### 3.4 Soil moisture

The zipped folder “SENTHYMED_MEDOAK_soil_moisture.zip” contains five files:

• SENTHYMED_MEDOAK_soil_moisture_data.txt

This file includes soil moisture and soil temperature probe measurements, with corresponding information on ID_LOC, MEAS_DATE, MEAS_TIME, MOISTURE_DEVICE_LAB, MOISTURE_DEVICE_TYPE, SITE, SITE_PLOT, ID_MEAS, repetition (for PSL only: between 3 and 4 measurements performed very close to the same location), depth (maximum depth of measurement in cm), volumetric soil moisture (%), soil temperature (°C), electrical conductivity (dS/m) and SHA/SUN (position of the measurement location relative to sun exposition with SHA: in the shade or SUN: in the sun). The ID_LOC naming is as follows: MEAS_DATE + “_” + SITE + “_” + SITE_PLOT + “_MEAS”+ ID_MEAS for PSL (ex: 20210608_PSL_22_MEAS1) or “_ICOS” for PUE (ex: PUE_CP1_MEAS_ICOS). Please refer to Table 1 for the used acronyms.

• SENTHYMED_MEDOAK_soil_moisture_coord_P.shp (data type: positions)

This file provides the location for each soil moisture measurement along with information on ID_LOC, XCOORD and YCOORD. Please refer to Table 1 for the used acronyms.

**Fig. 8:**
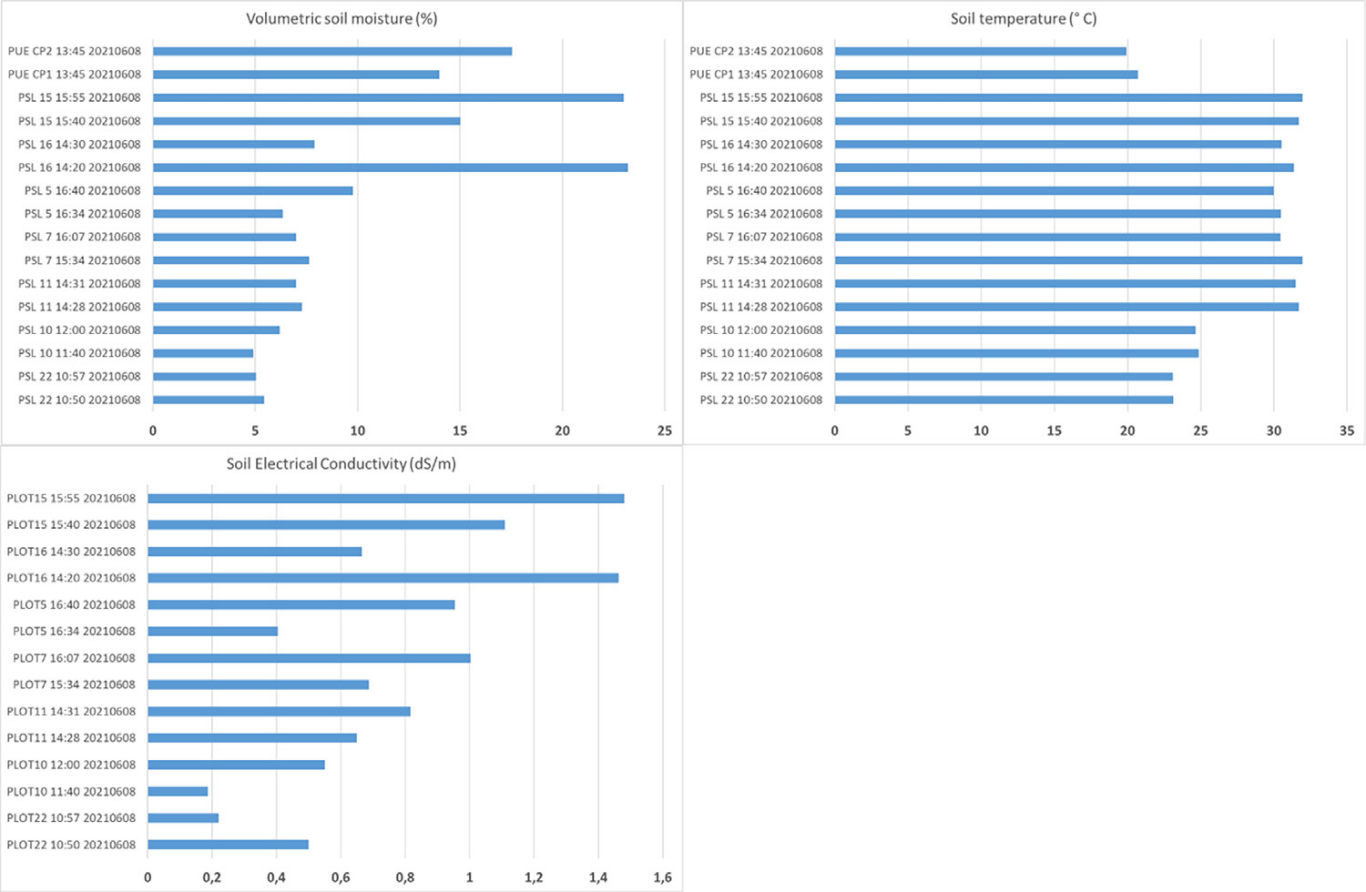
Soil moisture (top left), soil temperature (top right) and electrical conductivity (bottom) during the MEDOAK 2021 June campaign (for PSL: the average value per plot and calculated over the measurements, at-surface measures; for PUE: one measure per plot, measure at −5 cm depth).

• SENTHYMED_MEDOAK_soil_moisture_figure1.png (cf. Figure 8)

### 3.5 Leaf-clip optical sensor data

The zipped folder “SENTHYMED_MEDOAK_leafclip_optical_sensor.zip” contains three files:

• SENTHYMED_MEDOAK_leafclip_optical_sensor_data.txt

This file includes DUALEX and SPAD values along with ID_LOC, SAMPLING_DATE, SAMPLING_TIME, SITE, SITE_PLOT, SPECIES, TREE, TREE_LEAF, TREE_LEAF_AGE, LEAFCLIP_DEVICE_LAB, LEAFCLIP_DEVICE_TYPE, CHL_SPAD (non calibrated values that are correlated to leaf total chlorophylls content, without units, generally between 0 and 60), CHL_DUALEX (leaf total chlorophylls content in µg.cm^−2^), ANT_DUALEX (anthocyanins content, relative absorbance units, generally between 0 and 3), FLA_DUALEX (flavonoids content, relative absorbance units generally between 0 and 1.5) and NBI_DUALEX (Nitrogen Balance Index equaling the ratio CHL_DUALEX/FLA_DUALEX). The ID_LOC naming is as follows: SITE + “_” + SITE_PLOT + “_” + SPECIES + “_” + TREE (ex: PUE_CP1_QI_76). Please refer to Table 1 for used acronyms.

• SENTHYMED_MEDOAK_sampled_tree_coord_P.shp (data type: positions)

This file provides the location for leaf samples in the field (cf. Fig. 2), along with ID_LOC, XCOORD and YCOORD. Please refer to Table 1 for used acronyms.

**Fig. 9:**
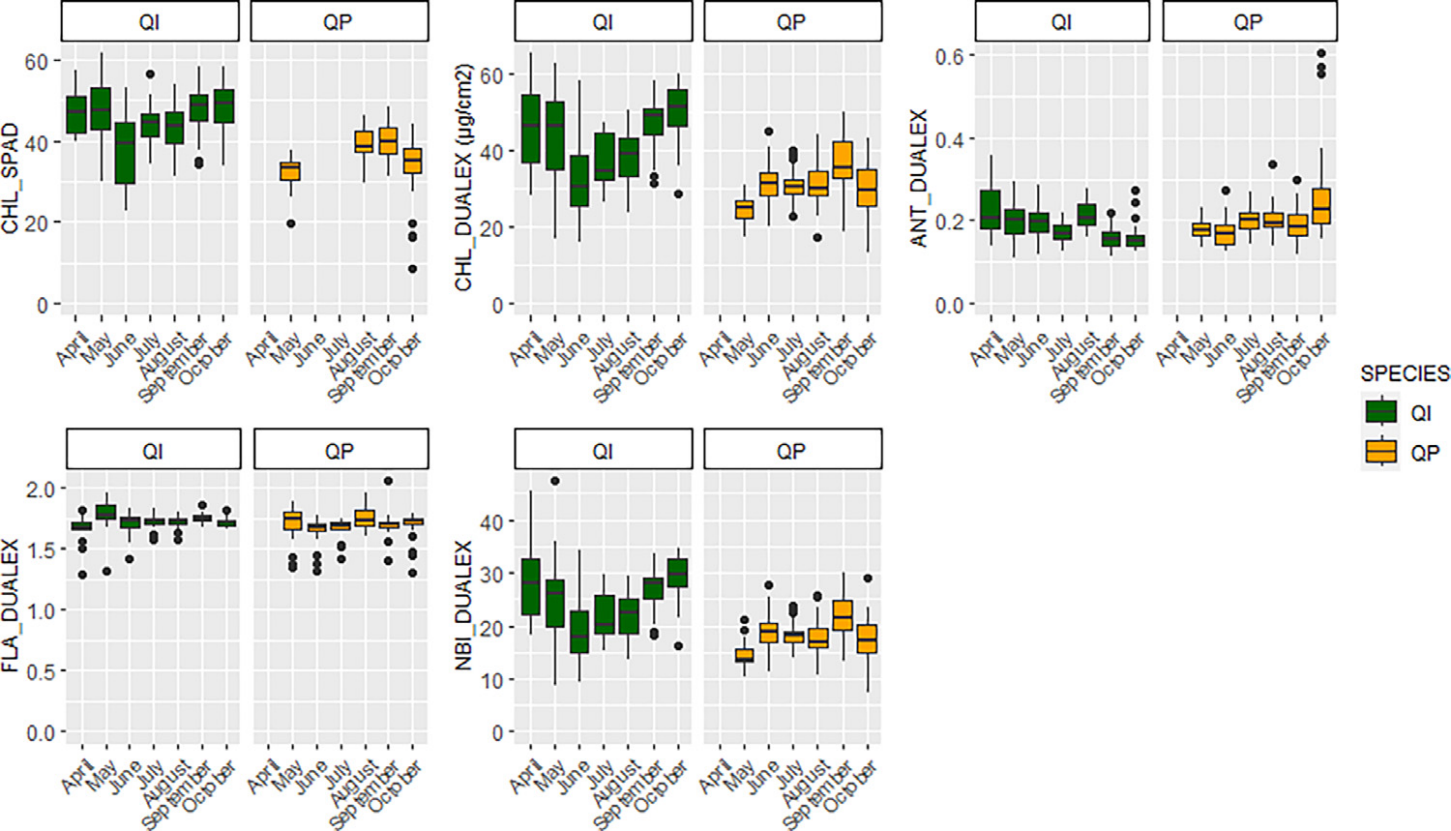
Phenological variations of the SPAD and DUALEX leaf-clip optical sensor measured variables represented in whiskers box (QP: orange, QI: green) for each sampling date (cf. Table 1 for used acronyms).

• SENTHYMED_MEDOAK_leafclip_optical_sensor_figure1.png (cf. Fig. 9)

### 3.6 Leaf optical properties

The zipped folder “SENTHYMED_MEDOAK_leaf_optical_properties.zip” contains four files:

• SENTHYMED_MEDOAK_leaf_optical_properties_data.txt

This file includes leaf directional-hemispherical reflectance and transmittance measured in the laboratory, along with ID_LOC, SAMPLING_DATE, SAMPLING_TIME, SITE, SITE_PLOT, SPECIES, TREE, TREE_LEAF, TREE_LEAF_AGE, SPECTRAL_DEVICE_LAB, SPECTRAL_DEVICE_TYPE, SPECTRAL_DEVICE_ACCESSORY, SPECTRAL_MEAS_TYPE, ID_MEAS, and leaf reflectance measured in the field with a contact probe and a leaf-clip, when applicable. Each column represents the reflectance or transmittance value for spectral bands from 350 nm to 2500 nm with 1 nm spectral sampling. The ID_LOC naming is the same as the previous subsection 3.5. Please refer to Table 1 for used acronyms.

**Fig. 10:**
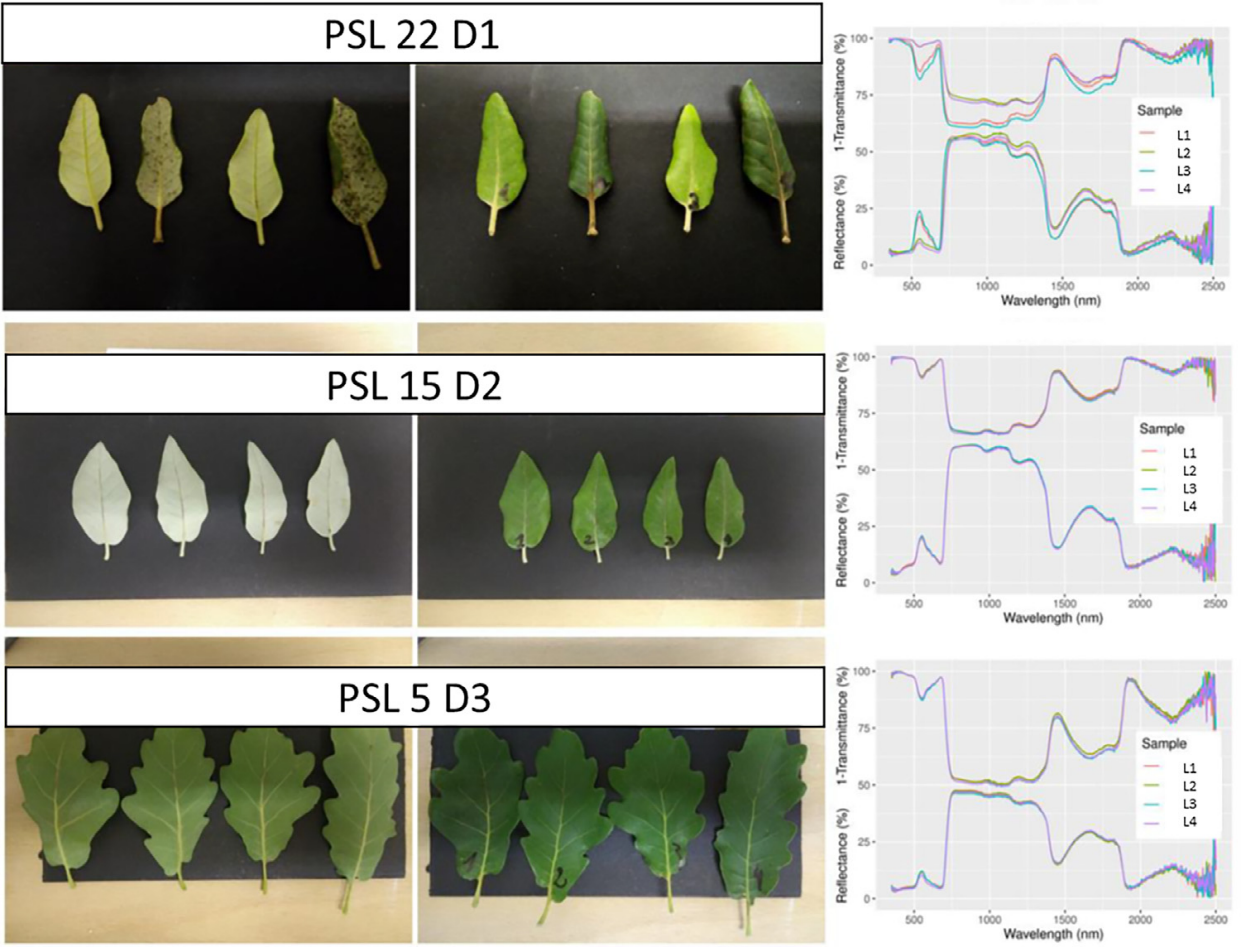
Leaf directional-hemispherical reflectance and transmittance measured in laboratory with an integrating sphere: QI with TREE_LEAF_AGE = Y for 2 leaves and O for 2 leaves (top row), QI with TREE_LEAF_AGE = O (middle row) and QP (bottom row). Please refer to Table 1 for used acronyms.
• SENTHYMED_MEDOAK_leaf_optical_properties_figure2.png (cf. Fig. 11)

• SENTHYMED_MEDOAK_sampled_tree_coord_P.shp (data type: positions, cf. subsection 3.5)
• SENTHYMED_MEDOAK_leaf_optical_properties_figure1.png (cf. Fig. 10)

**Fig. 11:**
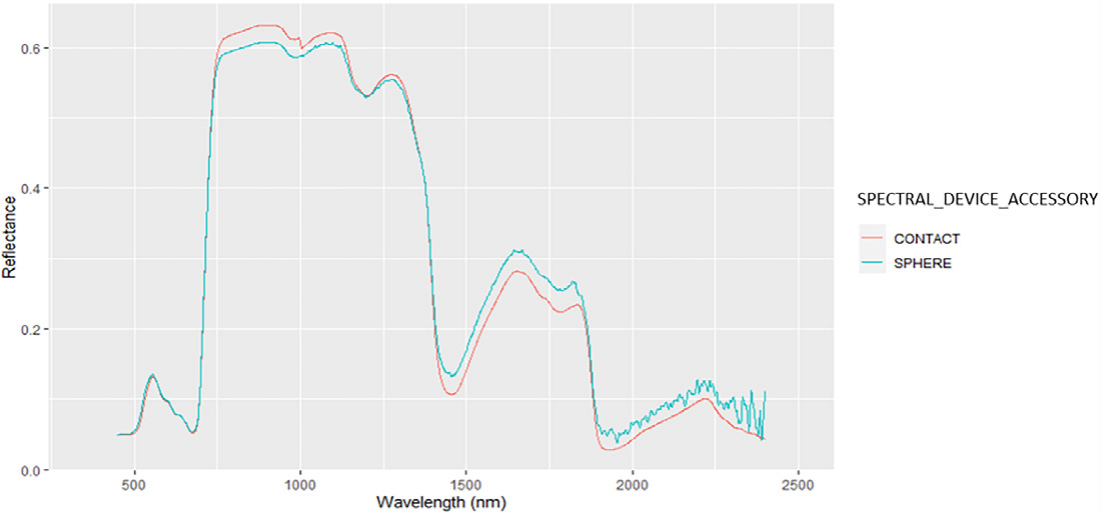
Comparison between directional-hemispherical reflectance measured with an integrating sphere (blue) and bidirectional reflectance measured with a contact probe (red) for a same QI leaf sampled in May 2021 (reference: PSL 10 D1 L1). Please refer to Table 1 for used acronyms.

### 3.7 Leaf trait data

The zipped folder “SENTHYMED_MEDOAK_leaf_traits.zip” contains five files:

• SENTHYMED_MEDOAK_leaf_traits_data.txt

This file includes leaf traits measured in the laboratory and corresponding estimates resulting from the inversion of the leaf radiative transfer model PROSPECT, along with ID_LOC, SAMPLING_DATE, SAMPLING_TIME, SITE, SITE_PLOT, SPECIES, TREE, TREE_LEAF, TREE_LEAF_AGE and LEAFTRAIT_LAB. Leaf traits include CHL_PROSPECT, CAR_PROSPECT and ANT_PROSPECT (total chlorophylls, carotenoids and anthocyanins content, µg.cm^−2^, obtained from PROSPECT inversion), EWT_PROSPECT and EWT_LAB (equivalent water thickness obtained from PROSPECT inversion and measured in the lab, mg.cm^−2^), LMA_PROSPECT and LMA_LAB (leaf mass area obtained from PROSPECT inversion and measured in the lab, mg.cm^−2^), PROT_PROSPECT and CBC_PROSPECT (nitrogen-based proteins and carbon-based constituents obtained from PROSPECT inversion, mg.cm^−2^). The ID_LOC naming is the same as the previous subsection 3.5. Please refer to Table 1 for used acronyms.

**Fig. 12:**
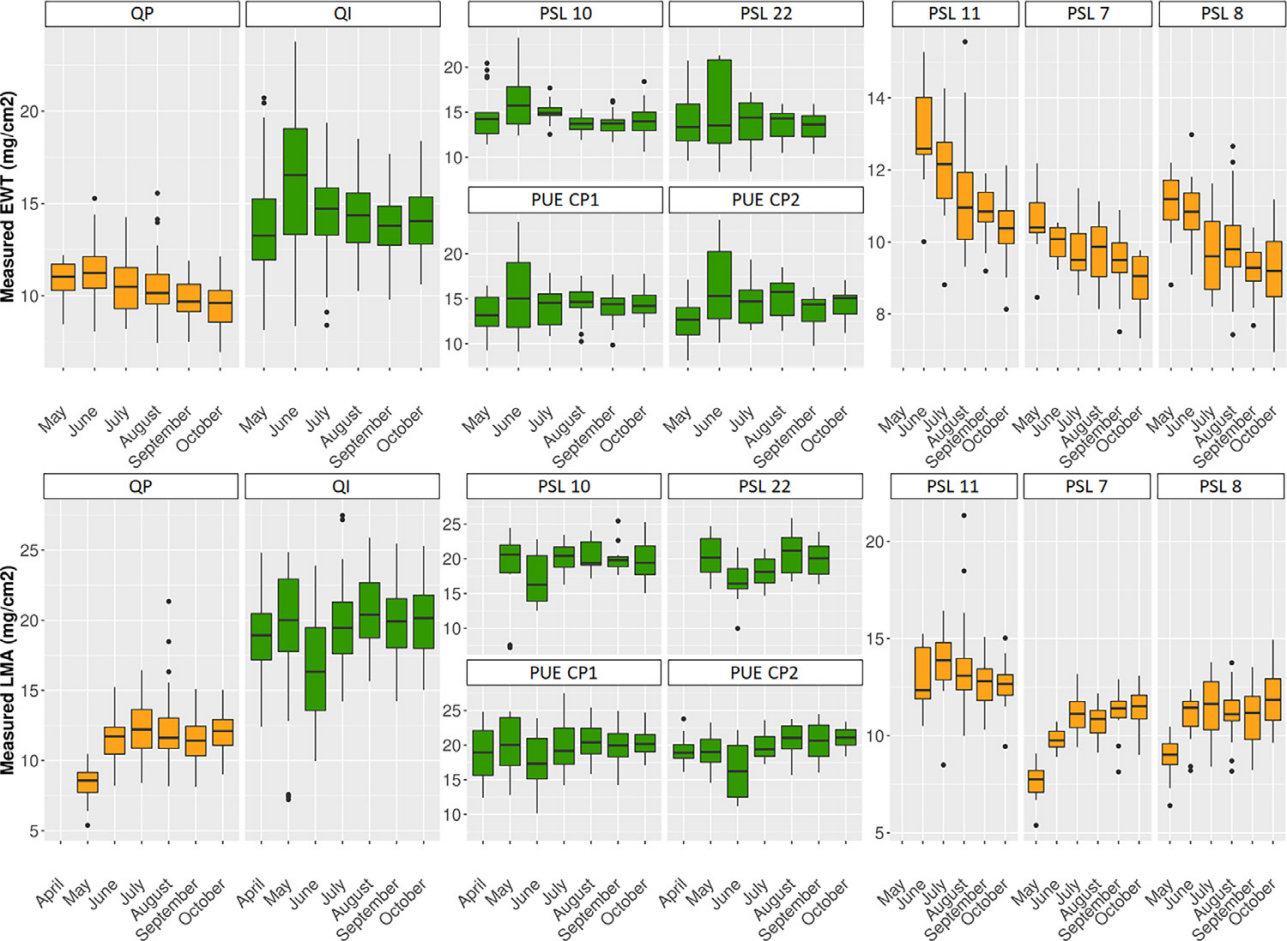
Phenological variations of the measured leaf traits represented in whiskers box (QP: orange, QI: green) for each sampling date. Note that for plot PSL 11, QP proportion represents 75% (i.e. 3 trees over the four samples). Please refer to Table 1 for used acronyms.
• SENTHYMED_MEDOAK_leaf_traits_figure2.png (cf. Fig. 13)

• SENTHYMED_MEDOAK_sampled_tree_coord_P.shp (data type: positions, cf. subsection 3.5)
• SENTHYMED_MEDOAK_leaf_traits_figure1.png (cf. Fig. 12)

**Fig. 13:**
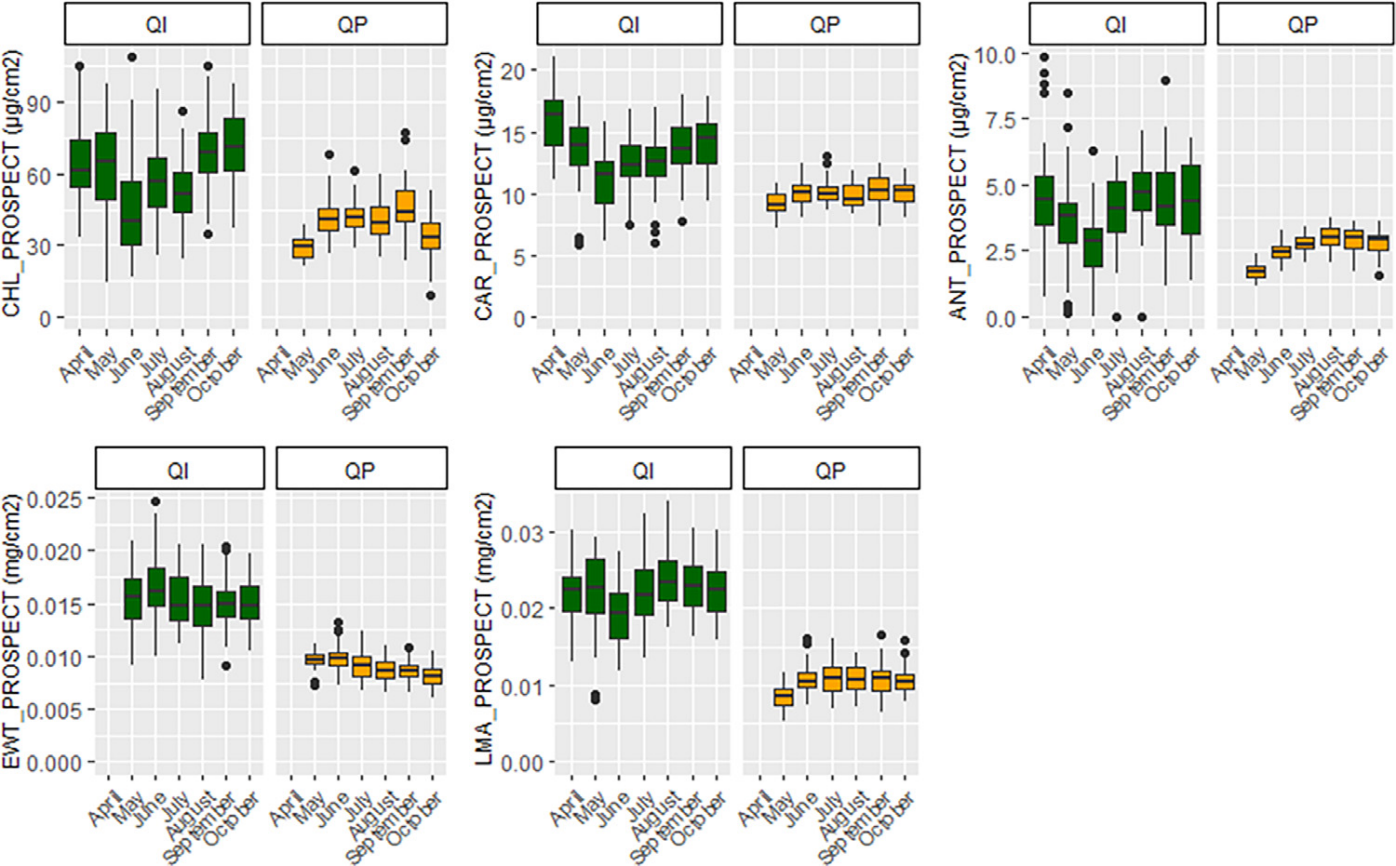
Phenological variations of the modelled leaf traits represented in whiskers box (QP: orange, QI: green) for each sampling date. Please refer to Table 1 for used acronyms.

### 3.8 UAV 3-D LiDAR data

**Fig. 14:**
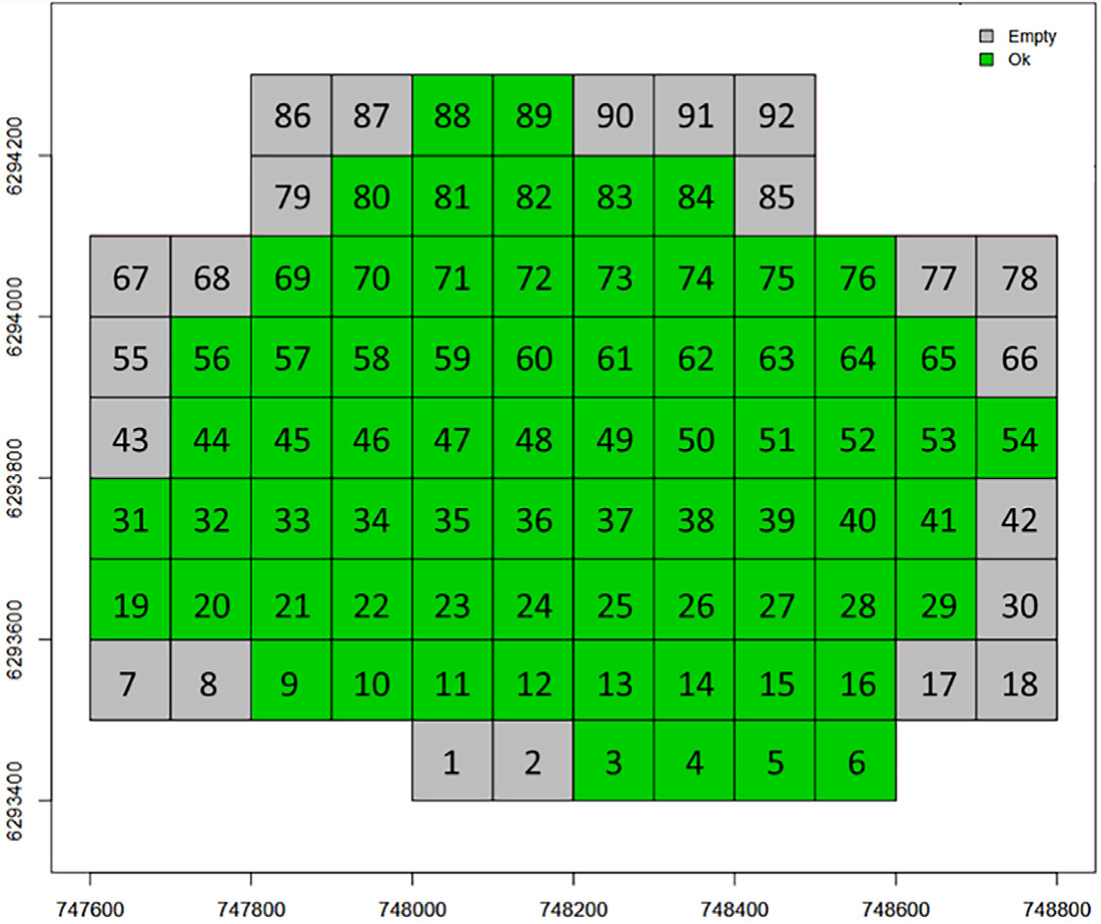
Grid of the 92 tiles scanning the coverage of PUE LiDAR acquisitions (cf. Fig. 20): tiles with no LiDAR data are in grey (essentially located at the borders) and those not empty are in green (figure not provided in the repository).

For PUE, the repository contains 70 files at the laz format (a compressed format of las file) derived from the use of LidR (R package). Each file corresponds to a tile of dimension 100 × 100 m^2^ embedded in a grid covering the full site coverage (Fig. 14). From a potential of 92 tiles, only 70 were not empty. For each tile, a number is attributed such as the file naming is as follows: “puechabon_” + number (between 3 and 89) + “.laz” (ex: puechabon_10.laz).

For PSL, the repository contains 4 files at the las format corresponding to the 4 tiles matching the 4 flight areas as depicted in Fig. 20. The file naming is as follows: “Strip-” + date of finished post-processing (format: YYYYMMDD, cf. SAMPLING_DATE in Table 1) + “-” + time of finished post-processing (format: HHMMSS, cf. SAMPLING_TIME in Table 1) + “_” + letter referring to one flight area (either A, B, C or D, cf. Fig. 20) + “.las” (ex: Strip-20210804-133525_B.las).

## 4 Experimental Design, Materials and Methods

This section is divided into three subsections. The first subsection describes the study sites and forest plots (4.1); the second subsection describes the protocol applied during data acquisition including the sampling strategy and instruments used (4.2); the third subsection provides information on airborne and satellite data acquired in the same time frame as ground data and completed with *in situ* calibration/validation activities (4.3).

### 4.1 Forest site and plot description

Two Mediterranean oak forest sites were selected, namely Puéchabon (PUE) and Pic Saint Loup (PSL), 17 km north of Montpellier, Occitanie Region, France.

PUE is located on a flat karstic limestone plateau inside the Puéchabon national forest that extends over several hundred hectares and is largely dominated by holm oak (*Quercus ilex*), which represents more than 90% of the forest cover. The site is characterized by low soil water reserve (approx. 140 mm), resulting in strong and recurrent water stress of vegetation during summer period. The SENTHYMED/MEDOAK measurements were taken in the CNRS concession of 54 hectares. The latter represents an experimental site managed by the CEFE laboratory since 1984 and is a reference site for many national and international projects and observatory networks such as CarboEurope-IP and FLUXNET, and European research infrastructures such as ICOS and AnaEE [6]. Long-term scientific studies include among others the continuous monitoring of carbon and water fluxes between the atmosphere and the forest, and the assessment of the ecosystem response to climate change and increased drought through rain exclusions and thinning experiments. Meteorological mean statistics indicate an annual precipitation of 916 mm and an average annual temperature of 13.2 °C. Additional data including meteorological variables, eddy covariance fluxes and soil moisture measurements acquired following the ICOS protocols are available on the ICOS data portal where PUE is referenced as FR-Pue [7].

PSL is a low altitude mountain range (max 658 m) which covers an area of 568.8 km², and is part of various protected areas (ex: Natura 2000 and ZNIEFF - natural zone of ecological interest for fauna and flora). It has undergone significant changes in land use over the past decades, notably due to the receding of pastoral, agricultural and viticultural activities. The vegetation is composed of holm oak (*Quercus ilex*), pubescent oak (*Quercus pubescens*), and Aleppo pine (*Pinus halepensis*). Pubescent oak, which is the most competitive species, should theoretically dominate the whole landscape. However, Aleppo pine (a species resistant to forest fire) and holm oak (a species resistant to forest cutting) are dominant species due to anthropization. Meteorological records from two nearby stations measured an average annual temperature of 13 °C and an average annual precipitation of 740 mm.

A survey carried out on February 2021 aimed at selecting circular plots of diameter 30 m × 30 m (in order to fit the maximum spatial resolution of the spectroscopic imaging data such as PRISMA, SBG and CHIME) over the two sites which encompass a large variability in terms of species composition and canopy cover fractions. The prospection relied on the BD Forêt® V2 raster by the French National Geographic Institute or IGN (https://geoservices.ign.fr/bdforet) giving information about areas where the forest was dense or open with only deciduous oaks, only evergreen oaks, or mixed. Over a total of 35 surveyed plots, 13 were retained for the project with characteristics given in Table 3: 2 on Puéchabon and 11 on Pic Saint Loup. The two selected plots on PUE had previously been inventoried between September and October 2020 following the ICOS protocol [8] (two plots of 25 m of diameter, named CP1 and CP2) with a total of 1500 sampled trees. The plot selection on PSL was done so as to avoid as much as possible plots with topography and the presence of conifers. For each plot, the mean elevation and the slope of the terrain were computed from BD ALTI® 25M raster by IGN (https://geoservices.ign.fr/bdalti) and the QGIS toolbox. The canopy cover was computed from the UAV LiDAR 3-D data with the lidR package, at the scale of 30 m side squares framing each circular plot [9]: (i) ground and non-ground points were distinguished by applying a multiscale curvature classification algorithm, (ii) digital elevation models (DEM) were then generated with the inverse distance weighting algorithm, (iii) canopy height models with a 25 cm spatial resolution were created by applying a triangulation algorithm to the LiDAR point clouds normalized by the DEM, and (iv) pixels with a height above 1 m were considered as part of the canopy. The species composition of each plot was computed from forest inventory, by calculating the crown area per species in relation to the total QI+QP area. Thus, inaccuracy may occur if the inventory is not complete on the plots.

**Table 3:** Characteristics of the selected forest plots over the two sites (cf. Table 1 for used acronyms).

SITE	SITE_PLOT	TREE	SPECIES	Species composition (QI%/QP %)	Canopy cover (%)	Elevation above sea level (units: meters)	Slope of the topography (units: %)
PUE	CP1	290, 452, 618, 76	QI, QI, QI, QI	∼100/0 ∼100/0	100%	271	5
	CP2	1113, 1151, 1411, 822	QI, QI, QI, QI		98%	272	5
PSL	4	NA	NA	40/60	90%	255	3
	5	D1, D2, D3, D4	QP, QP, QP, QP	50/50	92%	254	3
	6	NA	NA	10/90 10/90 10/90	NA	253	3
	7	D1, D2, D3, D4, D5	QP, QP, QP, QP, QP		82%	252	3
	8	D6, D7, D8	QP, QP, QP		92%	252	1
	10	D1, D2, D3, D4, D5, M4	QI, QI, QI, QI, QI, QI	∼100/0	71%	313	12
	11	D13, D3, D2, D10, D6	QI, QP, QP, QP, QP	40/60	100%	303	15
	14	NA	NA	NA	NA	262	7
	15	D1, D2, D3, D4, D5	QP, QI, QP, QP, QI	NA	NA	266	4
	16	D1, D2, D3, D4	QP, QP, QP, QI	NA	NA	264	5
	22	D1, D2, D3, D4	QI, QI, QI, QI	∼100/0	98%	363	26

On each plot, between 4 and 5 dominant trees were selected (crown size visible from the top of canopy and so from remote sensing images) following the tree species composition and scanning the best the plot area, when possible (cf. Fig. 2 and Table 3).

### 4.2 Data acquisition

#### 4.2.1 Generalities

Each 2021 monthly campaign consisted of 2–3 days with 2 days dedicated to fieldwork and 1–2 day dedicated to laboratory work, except the MEDOAK campaign for which additional data were collected. In general, the first day was dedicated to forest inventory in the afternoon and PAI measurements at sunset for PSL. The second day was dedicated to leaf sampling, measures with leaf-clip optical sensors, leaf conditioning in the morning and beginning of afternoon, and when possible *in situ* spectral measurements in the afternoon. Then the same day, the conditioned leaves were carried out to the TETIS laboratory in Montpellier for leaf spectral and trait measurements in the afternoon (sometimes also the day after). Therefore, the time elapsed between leaf sampling and measurements was usually less than 8h.

The dates of the seven monthly campaigns over the April-October 2021 period were ruled by Sentinel-2 overpass dates, to span ideally the full phenological cycle of *Quercus pubescens* from leaf sprouting until leaf senescence. In order to avoid a posteriori uncontrolled uncertainties related to the choice of either the Sentinel-2A or 2B sensor (different radiometry and geometry inducing inter-sensor post-processing problems), it was preferred to fix the dates only for a single sensor overpass, here 2A. And so, leaf sampling dates as well as PAI measurement dates at PSL were distant to Sentinel-2A overpass ones by one day, except June and October (Table 4). For PUE, PAI measurements were taken during the already on-going ICOS LAI campaigns in order to optimize and reduce the field work load (Table 4). Soil moisture in PUE was continuously recorded at several depths according to the ICOS protocols, while in PSL soil moisture was only measured during the MEDOAK fieldwork in June. The April campaign was a test campaign in order to verify the measurement protocols deployed and their feasibility in the field. The six other campaigns corresponded to operational field campaigns. The additional June 2023 field campaign targeted only the refinement of forest overstory inventory, the achievement of the understory inventory and few complementary spectral measurements. Forest overstory inventory for the PUE plots were not provided in the SENTHYMED/MEDOAK data since they are already available through the ICOS data portal [7]. The AVIRIS-Next Generation flights were originally planned for the 8th of June 2021, but there were postponed to the 9th and 10th of June for lack of airport authorization. However, the leaf data collection at PSL and PUE as part of the MEDOAK field campaign were maintained on the 8^th^ because few human resources were available the following days.

**Table 4:** Summary of acquisition days between the monthly field campaigns and the multi-platform optical remote sensing data over the year 2021 (Sentinel-2 days highlighted in bold are those taken as references to fix the monthly dates, satellite days highlighted in italics are for cloud cover less than 30% at tile scale). Grey boxes mean no data collection.

These campaigns involved up to 19 people (researchers, PhD students, interns, contractor engineers, etc.) and on average between 6 and 13 people on the eight campaign dates. Each protocol for data collection and measurement described in Section 3 is detailed hereafter. A summary of data collection is given in Tables 4 and 5.

**Table 5:** Detailed summary per plot and site of the performed measurement types for the 2021 monthly field campaigns (leaf sampling/leaf-clip & optical property & biochemistry data: x symbol, PAI data: circle symbol, soil moisture data: star symbol, LiDAR 3-D data: plus symbol) and global summary of forest inventory by including June 2023 field campaign (last row, forest overstory: diamond symbol, forest understory: plus symbol). Grey boxes mean no data collection.

#### 4.2.2 Forest inventory

Four GPS instruments were used to record the geographical coordinates of the collected data: three Trimble instruments (GeoExplorer 6000 series, Geo7X series and Juno T41/5) using the GPS Pathfinder office software, and one SparkFun RTK Surveyor with Drotek DA910 antenna and SW Maps application using Centipete RTK network. All supplied files were created from QGIS software. The understory inventory was performed only during the 2023 June field campaign while the overstory one is a concatenation of data collected over all the field campaigns in 2021 and 2023.

**Fig. 15:**
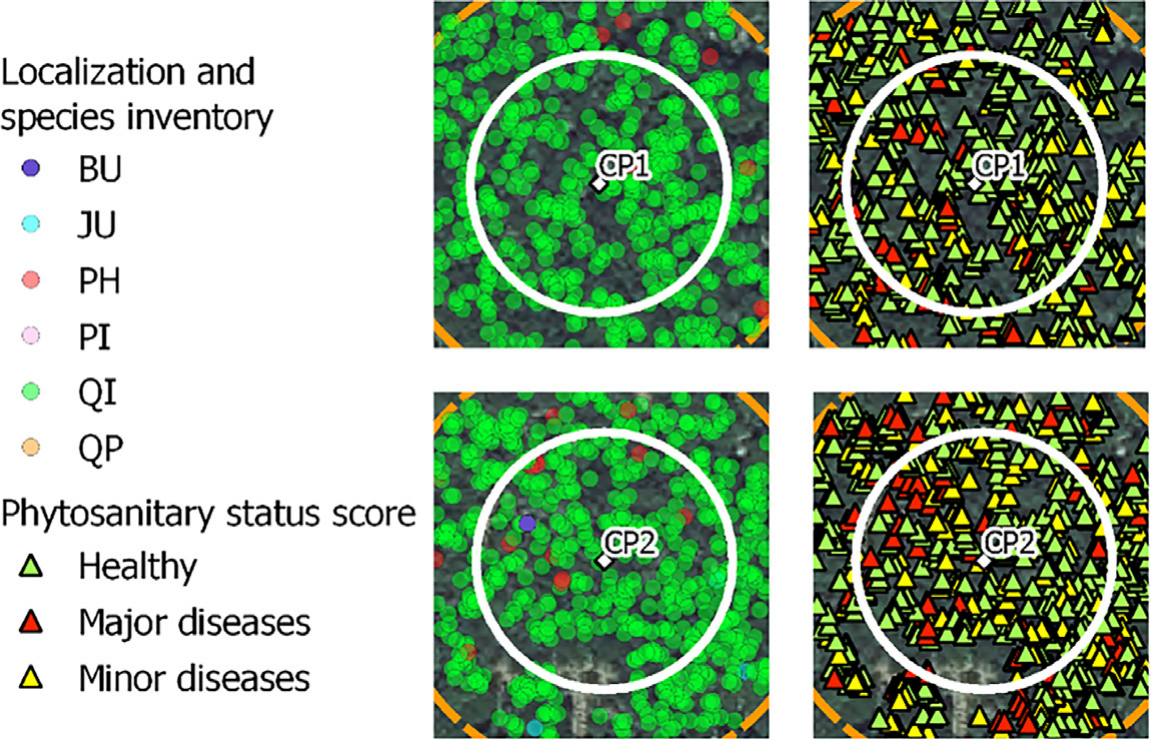
ICOS inventory on the two plots of PUE in 2020 (cf. Table 1 for used species acronyms).

For the overstory and for one given tree, both the tree trunk position and the tree crown area were recorded when possible. The crown area is reported as seen from top of canopy. This measurement provides more uncertainties when the canopy cover is very dense and the tree density very high, such as in plot 11 and 22. This inventory type is almost complete for plot 4, 5, 6, 7, 8, 10 and partially done for 15 and 16 where less data were collected (cf. Figs. 2 and 3). Systematically, the sampled trees were at least done, and globally, four vegetation species were inventoried (QI, QP, PI, JU). For PUE and as already mentioned, a very precise inventory is already available for plots CP1 and CP2 on the ICOS portal [7](Fig. 15).

For the understory and for one given plot, the on-ground inventory was performed on all the twenty-five locations of the “snake pattern” sampling grid defined for the PAI measurements at PSL (see following subsection 4.2.2, ID_MEAS from 1 to 25). A total of twelve surface types were identified with a majority of MIXLIL and MIXGLIL, and in a lesser proportion, LIMESTONE and MIXGL (Figs. 3 and 4).

#### 4.2.3 Canopy plant area index

**Fig. 16:**
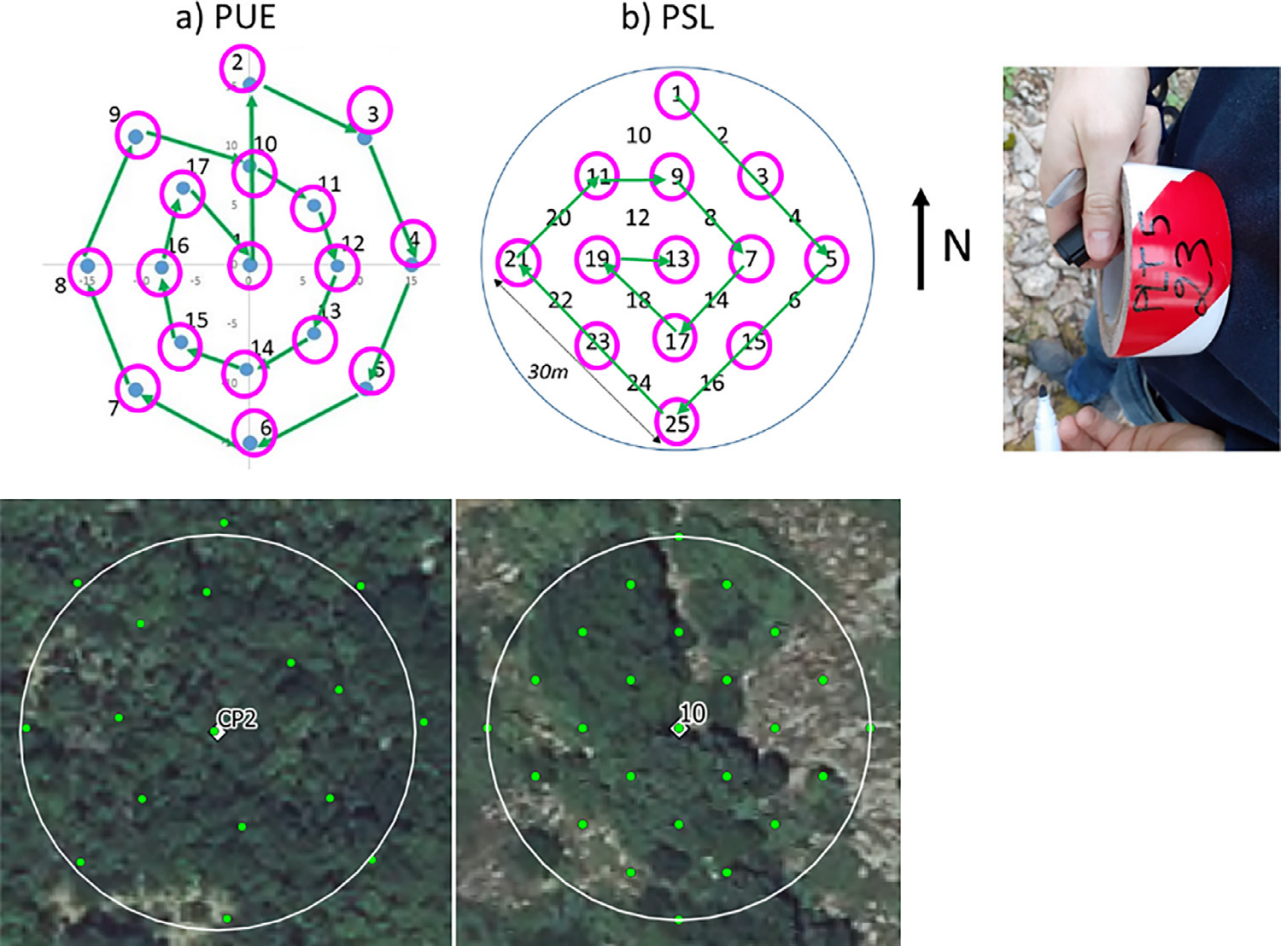
PAI sampling grids for a) PUE (with CP2 plot illustration from QGIS software) and b) PSL (with plot 10 illustration) with sampled locations indicated in magenta circles and measurement order path indicated with green arrows, and photo of a ribbon tape used to mark the locations (example for plot 5 ID_MEAS 23).

PAI measurements were performed either with one LAI-2200 or two LAI-2000 plant canopy analyzers (LI-COR Biosciences) in the photosynthetically active radiation range between 400 nm and 700 nm. In either case, the two-sensors mode was used to perform the measurements, with one optical sensor measuring clear sky in the open and the second sensor measuring below the canopy. Before starting any measurement, the two optical sensors were matched together following the Li-COR user manual procedure to make sure that they give the same readings when measuring clear sky. All the measurements were performed exclusively either at dawn or at sunset (with a majority at sunset) with a view cap of 270° on the optical sensors to restrict the measurement field of view in the direction of the operator and with the field of view oriented in the East direction (when measurements were performed at sunset) to avoid as much as possible direct sunlight. The above canopy measurements were recorded in an open area located close to the plots for PSL and on a scaffolding platform for PUE while the below canopy measurements followed a sampling grid on each plot derived from the ICOS protocol [8](Fig. 16):

• For PUE: a “cross pattern” made originally for digital hemispherical photography measurements performed on circular plots (named CP) was adapted. A total of seventeen locations were sampled with an increasing measurement order from 1 to 17. If enough time was allowed, a last measurement was performed on the first location at the plot center (here 1) with the objective to compare if the retrieved PAI values are the same or very close between the beginning and the end of the plot measurements. A total of seventeen records per plot minimum was then acquired.
• For PSL: a “snake pattern” made originally for ceptometer measurements composed of twenty-five locations was used. Because only one day per monthly campaign was dedicated to the PAI measurements and the goal was to sample multiple plots, the sampling grid was reduced with one point over two for a total of thirteen locations by conserving a homogeneous sampling grid. Thus, the measurement order was 1-3-5-15-25-23-21-11-9-7-17-19-13. Thirteen records per plot were recorded in total.

Ribbon tape was used to mark the locations on each plot and to perform continuous measurements on the same locations for all field campaigns (Fig. 16). The first day of each campaign, checking was done if the re-establishment of these markings was necessary due to disturbances caused by weather events or wild animals.

The merging of the data acquired by the two optical sensors and the effective PAI computation (as well as the five gap fraction values computed for the five optics given five different zenithal orientations) were performed using the FV2200 2.1.1 software (2010 LI-COR Biosciences) and following the procedure described in the user manual. Files with above and below canopy readings were imported and merged by pairing the closest in time above canopy reading to each below canopy measurements (time lag was always less than 30 s which was the above canopy acquiring frequency). We used the horizontal default canopy model proposed by the FV2200 software and discarded measurements when the light transmittance below canopy was higher than 100%. Some computed effective PAI values were removed from the data because their values were too high (comprised between 4 and 11 m^2^.m^−2^ for September acquisitions on PSL plot 8 and PUE CP1). Also, some LAI-2000 data were identified as bad readings but kept in the supplied data. It mainly concerns plot 10 for some recurrent positions over the sampling dates (ID_MEAS equaling 5 and 23). It is worth noting that the PAI reported in the datasets is not the true PAI but the effective PAI [10].

#### 4.2.4 Forest optical properties

Spectroscopic measurements of natural surfaces (except leaves) and dirt roads were performed mostly outside of the studied plots in open parts visible from the remote sensing images as much as possible. Bi-directional reflectance spectra in the range 0.35–2.5 µm and with a 1 nm spectral step were acquired from two spectroradiometers: an ASD (Analytical Spectral Devices, Boulders, CO, USA) FieldSpec 3, with a spectral resolution of 3 nm from 0.35 to 1 µm, and 10 nm from 1 to 2.5 µm, and an ASD FieldSpec 4 Hi-Res with a spectral resolution of 3 nm from 0.35 to 1 µm, and 8 nm from 1 to 2.5 µm. The two instruments were not inter-calibrated. Standard protocols were followed. Remote measurements were performed at a distance of 1 m from the target surface with the bare optic fiber fixed in the gun and with/without fore optics to constrain the field of view. The operator wore dark clothes and positioned perpendicularly to the sun direction to avoid as much reflection disturbances as possible. Clear sky conditions were preferred but not always fulfilled. In any case, several calibration measurements were acquired with a white reference Spectralon® of known reflectance. For instance during the 2021 May campaign at PUE, the sky was overcast, however the measurement quality was visually checked as good. Same calibration measurements were done for those performed with the contact probe, with particular attention to avoid interference from sunlight with the interior probe light source illumination. Generally, a sequence of about 10–20 acquisitions was configured, leading to the same number of output files giving reflectance spectra. Each acquisition resulted from an average of 10 to 20 acquisitions. Each surface was only measured once without repetition. During the 2021 June campaign, spectral artefacts were reported in the 2–2.5µm region when using the ASD FieldSpec4 Hi-Res instrument, possibly due to too short warming stage. Attention should be paid when using these spectra.

#### 4.2.5 Soil moisture

**Fig. 17:**
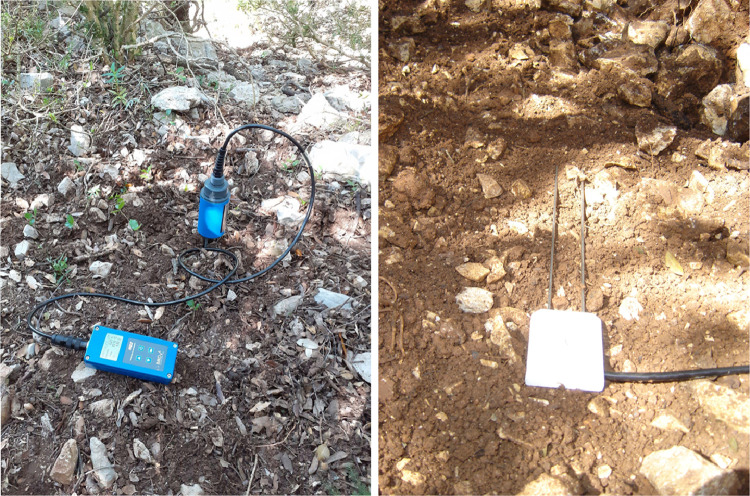
Photos of soil moisture measurements at PSL on plot 22 (left, photo by Karine Adeline, ONERA) and at PUE (right, photo by Jean-Marc Limousin, CEFE).

In PUE, volumetric soil moisture content was continuously recorded with Campbell CS616 soil moisture probes and soil temperatures with PT100 sensors logged onto a Campbell Scientific data logger (Fig. 17). Measurements were taken at three depths (−5 cm, −10 cm and −20 cm) according to the ICOS protocols. Measured values were recorded every half hour and averaged between 11 h00 and 16 h30 for the days when leaf samples were collected. In PSL, volumetric soil moisture content, soil temperature and electrical conductivity were measured with a 2-pin IMKO HD2 TDR (time-domain reflectometry) probe. These measurements were punctual and carried out only during the MEDOAK campaign over two days, the 8th and 9th of June. They were done for seven plots in PSL (22, 16, 15, 11, 10, 7 and 5) with two measurement locations per plot. For each location, measurements were repeated between three and four times and done side by side to have an estimation of the spatial variability. For the total of fourteen measurements done between 10 h50 and 16 h40 (local time), only one was in the sun while the others were in the shade due to the spread of the oak canopy. The terrain was poorly conducive to this kind of measure because on-ground understory was mainly composed of outcropping limestone rock stones with very little soil at the surface. However, when the latter was present, it was very difficult to penetrate the probe pins to a sufficient depth in the soil, that is why the penetration depth was recorded for each measurement (Fig. 17).

#### 4.2.6 Leaf sampling, *in situ* measurements and conditioning

**Fig. 18:**
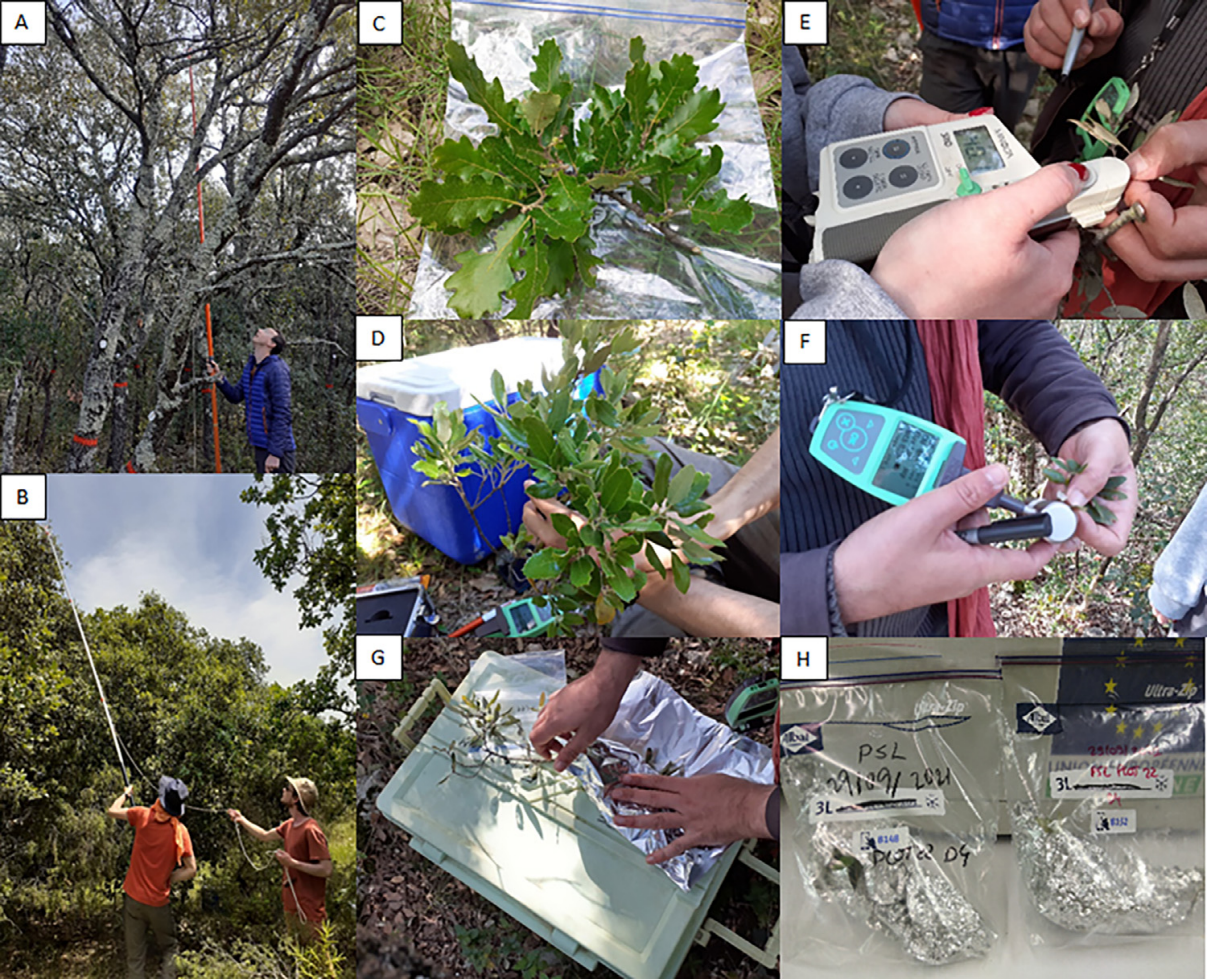
Photos A and B illustrate leaf sampling at PUE and PSL respectively; photos C and D represent the sampled twigs for QP and QI respectively; photos E and F illustrate measurements with the SPAD and DUALEX respectively; photos G and H illustrate leaf conditioning (photos by Karine Adeline, ONERA).

From each sampled tree, a single twig was cut at the sunlit treetop canopy with a pruning shear attached to the end of a telescopic pole that could be operated using a rope (Fig. 18). The maximum height of 6 m is reached with the pruning shear, which was sufficient for QI but not systematically for QP. Four representative leaves were selected from each twig. The proportion of new and old generation leaves was visually assessed for QI and accounted for when sampling leaves. Each leaf was marked by a number between one and four with a permanent marker. For each leaf, a single measurement was performed by one or two of the following leaf-clip optical sensors: DUALEX-4 (Force-A, Orsay, France) and SPAD-502Plus (Konica-Minolta, Tokyo, Japan)(Fig. 18). During the May 2021 campaign, additional spectroscopic measurements were performed with a contact probe for each leaf for further comparison with those performed later in the laboratory (cf. Fig. 11). A paper towel soaked in water was wrapped around the base of the twig. The latter was wrapped in aluminium foil to avoid sunlight contact and placed in a ziplock bag marked with a unique identifier including SAMPLING_DATE, SITE, SITE_PLOT and TREE)(Fig. 18, cf. Table 1 for used acronyms). The ziplock bag was stored in a cooler containing ice packs. The cooler was then transferred to the lab facility in Montpellier the same day for the next measurements.

#### 4.2.7 Laboratory leaf measurements

**Fig. 19:**
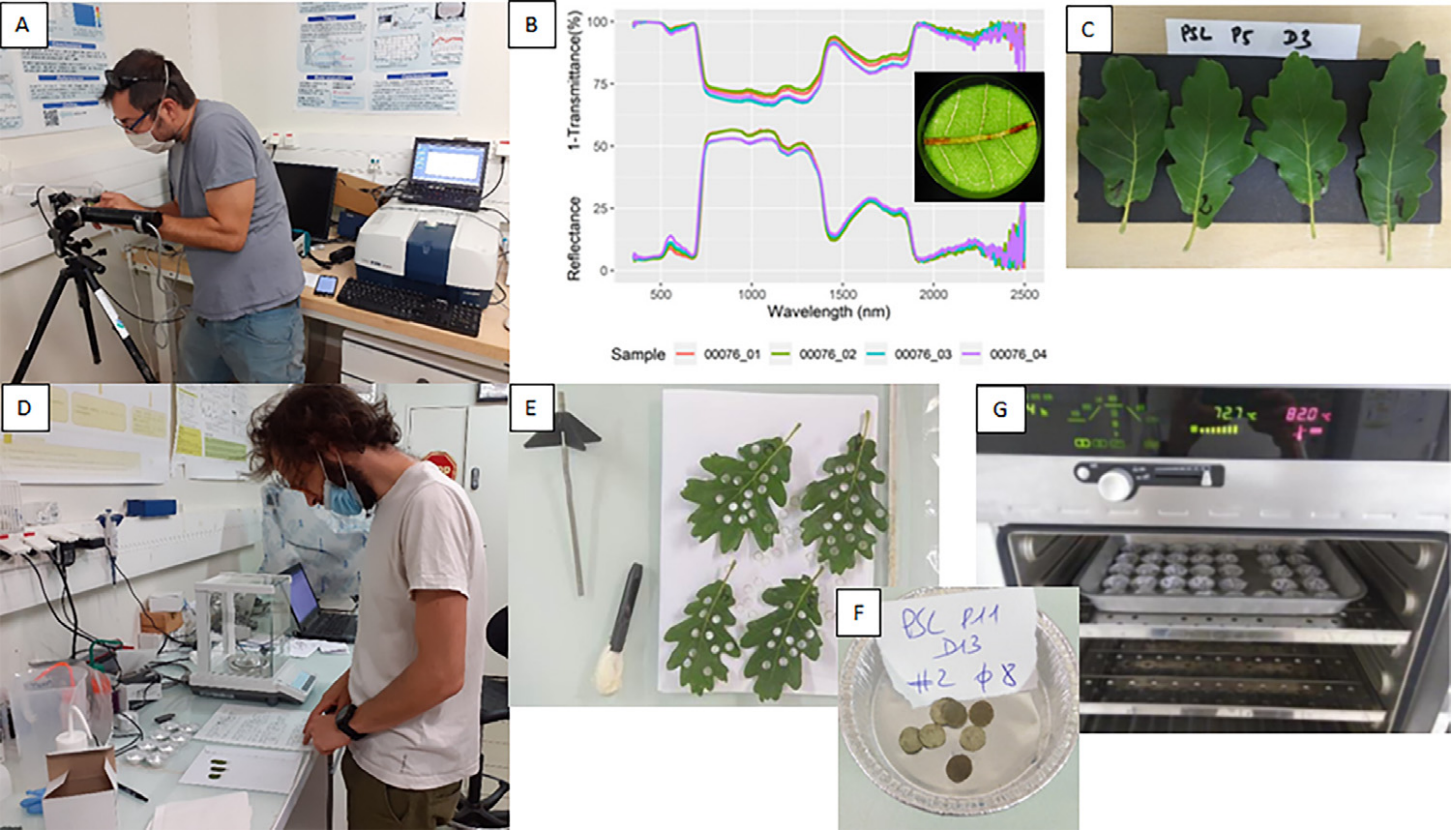
Photos A, B and C from the spectral measurements of the four leaves, photos D, E, F and G from the leaf weighting, punching and drying (photos by Karine Adeline, ONERA, Josselin Giffard Carlet and Jean-Baptiste Féret, INRAE).

Once in the lab facility, the ziploc bags containing the leaf samples were stored in a refrigerator. Then, optical properties were performed for each leaf sample. The set up for spectral measurements included an ASD Fieldspec 3 spectroradiometer coupled with a LiCor 1800-12S (LI-COR, Inc., Lincoln, NE, USA) integrating sphere coated with Barium sulphate, including a halogen light source (20 W, 2800 K)(Fig. 19). First, the stray light was measured in order to correct the next measurements. Second, a white reference was performed before each individual measurement (reflectance or transmittance). Third, reflected or transmitted light intensity were measured, and converted into directional-hemispherical reflectance and transmittance measurement, accounting for white reference and stray light as described in the instruction manual of the manufacturer available online (https://licor.app.boxenterprise.net/s/c0vkjb20o7r4lekvm21p). Once its optical properties were measured, the sample was placed between two wet paper towels to avoid drying out. Several circular subsamples were collected with a 6 mm round punch, avoiding the midrib of the leaf and necrotic leaf areas. Their fresh weight of the subsamples was measured by putting them into an aluminium cup and by using a 100 µg precision electronic balance. Each cup was placed in an oven set at a temperature of 80 °C for 48 h to 72 h. The weight of the subsamples was measured again to obtain the dry weight (Fig. 19). Equivalent water thickness (EWT) was computed as the difference between fresh and dry weight divided by the total leaf area while leaf mass per area (LMA) was obtained as the ratio between dry weight and total leaf area. The latter was retrieved from the total number of 6 mm diameter circular subsamples.

The leaf radiative transfer models PROSPECT-D [11] and PROSPECT-PRO [12] were then inverted by using an iterative optimization applied to reflectance and transmittance, as described in the tutorials available from the R package prospect available in [13]. PROSPECT-D inversion allowed assessing leaf pigment content (chlorophylls, carotenoids and anthocyanins), EWT and LMA, while PROSPECT-PRO allowed assessing nitrogen-based proteins and carbon-based constituents, two specific groups of constituents contributing to LMA.

#### 4.2.8 UAV-borne LiDAR 3-D point clouds

All LiDAR overflights by drone were performed in June 2021. The LiDAR sensor used was a Yellowscan Surveyor and was embarked on a DJI Matrice 600 Pro UAV. The YellowScan Surveyor includes:

• GNSS-inertial station : Applanix APX-15 UAV
• LiDAR : Velodyne VLP16 (also known as Puck) : Wavelength: 905 nm / 300,000 pulses per second (300 kHz) / 2 echoes per pulse / Viewing angle: 360° - Accuracy: 4 cm

The design of a flight path consists of a double grid with interline distances of 40 m. The drone flew at 50 m height constrained by a 5 m resolution DTM from the BD ALTI® by IGN. The flights were made at a speed of 5 m per second. All trajectories were planned with the UGCS-4.0.134 software. Then, the LiDAR dataset was post-processed in several steps by using different softwares:

• Applanix POSPac UAV 8.4: post-processing of trajectories based on the UAV's GNSS-Inertial Measurement System data and using a reference GNSS base station. The correction solution for each trajectory is exported in an ASCII SBET (Smoothed Best Estimated Trajectory) file.
• Yellowscan CloudStation V2106.0.0: The SBET file is integrated in the software to generate point clouds in .las format projected in RGF93/Lambert 93.
• LidR (R package): reading of las files (cf. Table 6), redrawing into tiles in compressed format .laz file corresponding to the output format.

**Table 6:** LiDAR raw and tiled data description for PUE and PSL (cf. Table 1 for used acronyms).

SITE	Zone	MEAS_DATE	Number of points (millions)	Density (point / m^2^)	Area (ha)	XCOORD min	XCOORD max	YCOORD min	YCOORD max
PUE	1	20210616	65.48	333.02	19.66	747695.4	748257.4	6293510	6294050
	2	20210616	33.35	311.83	10.69	748199.3	748588	6293410	6293803
	31a	20210616	38.66	196.36	19.68	747859.8	748420.2	6293690	6294219
	31b	20210616	26.86	173.1	15.51	747933.3	748426.7	6293711	6294191
	4	20210616	54.11	275.57	19.63	748153.3	748709.9	6293563	6294072
	5	20210616	24.34	225.29	10,8	748024	748403.1	6293473	6293881
	Final tiled data	20210616	242.79	449.4	54	747695.4	748709.9	6293410	6294219
PSL	A	23/06/21	27.73	246.9	11.23	762586.3	762996	6296938	6297296
	B	25/06/21	60.64	298.22	20.33	761207	761748.2	6295754	6296348
	C	23/06/21	33.13	223.19	14.84	763486.2	764065.2	6297102	6297465
	D	23/06/21	15.9	199.92	7.9	763619	763954.5	6297279	6297585

**Fig. 20:**
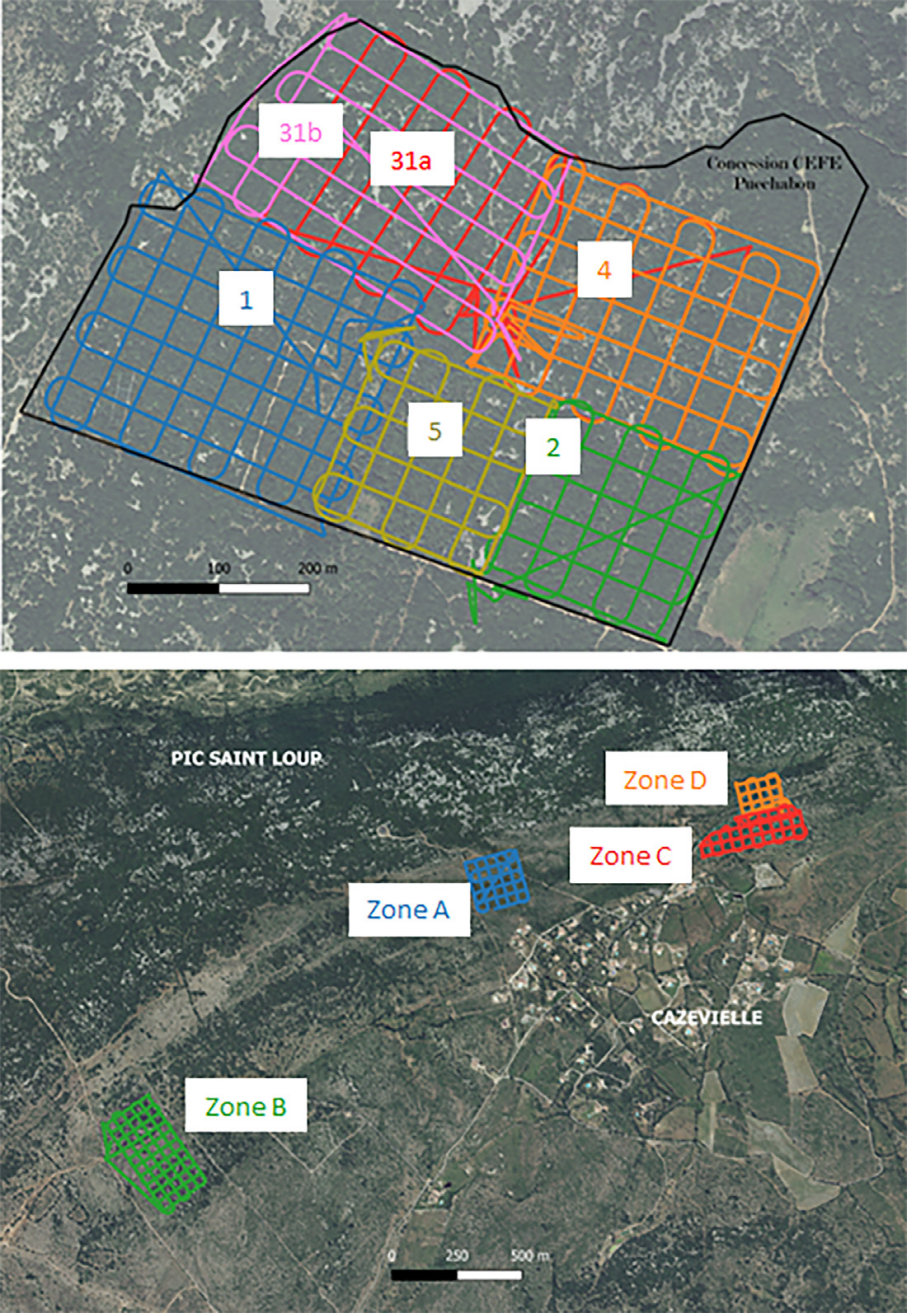
Top row: the 6 drone flights (1, 2, 31a, 31b, 4 and 5) over PUE. Note that the flight 31 was realized in two times because of a technical problem encountered during the first flight; Bottom row: the 4 drone flights (A, B, C, D) over PSL.

**Fig. 21:**
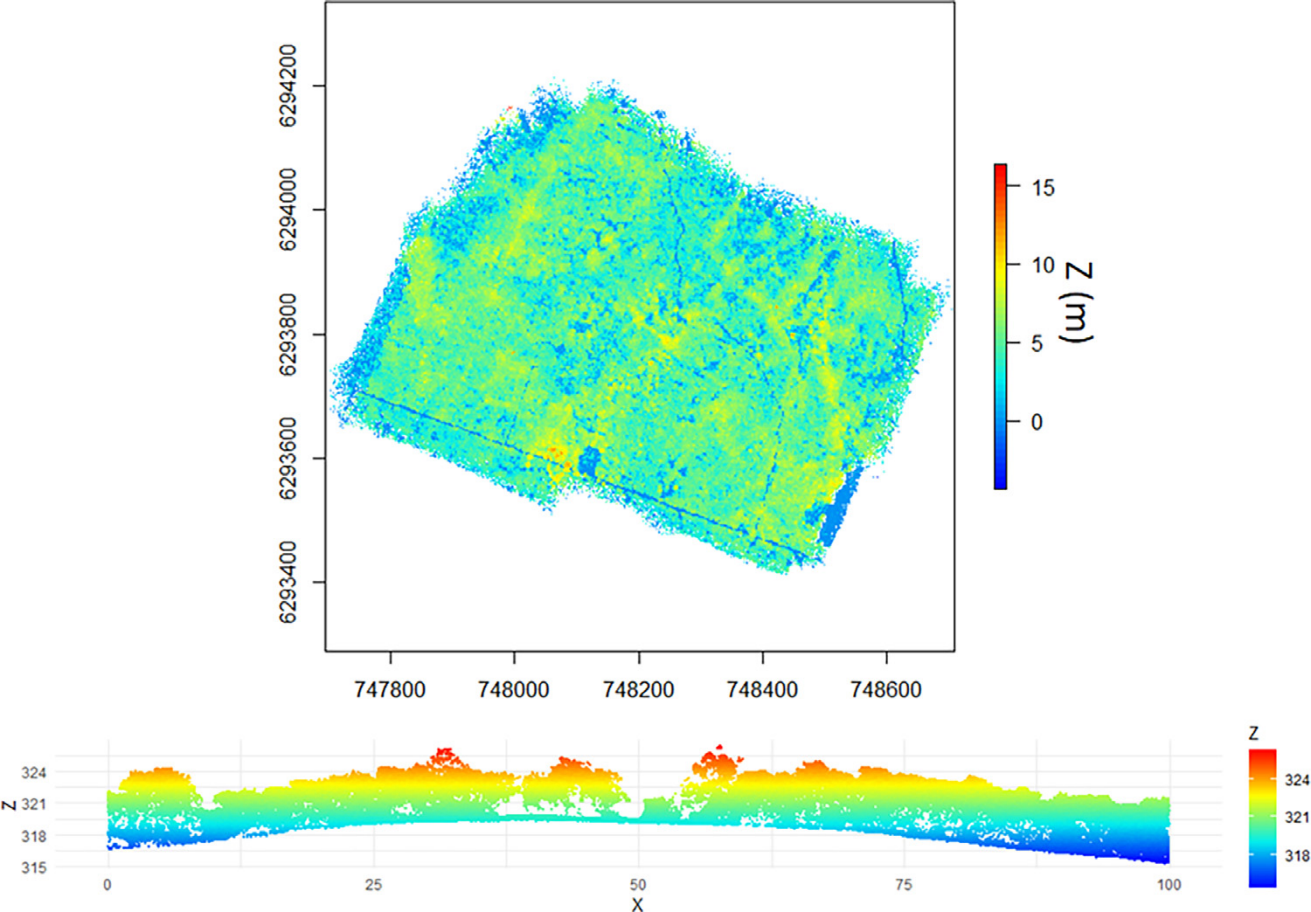
Top: cloud points from the final tiled data for PUE (Z: height above the surface); Bottom: cross section of LiDAR data over PUE (Z and X in meters, Z: height above sea level).

**Fig. 22:**
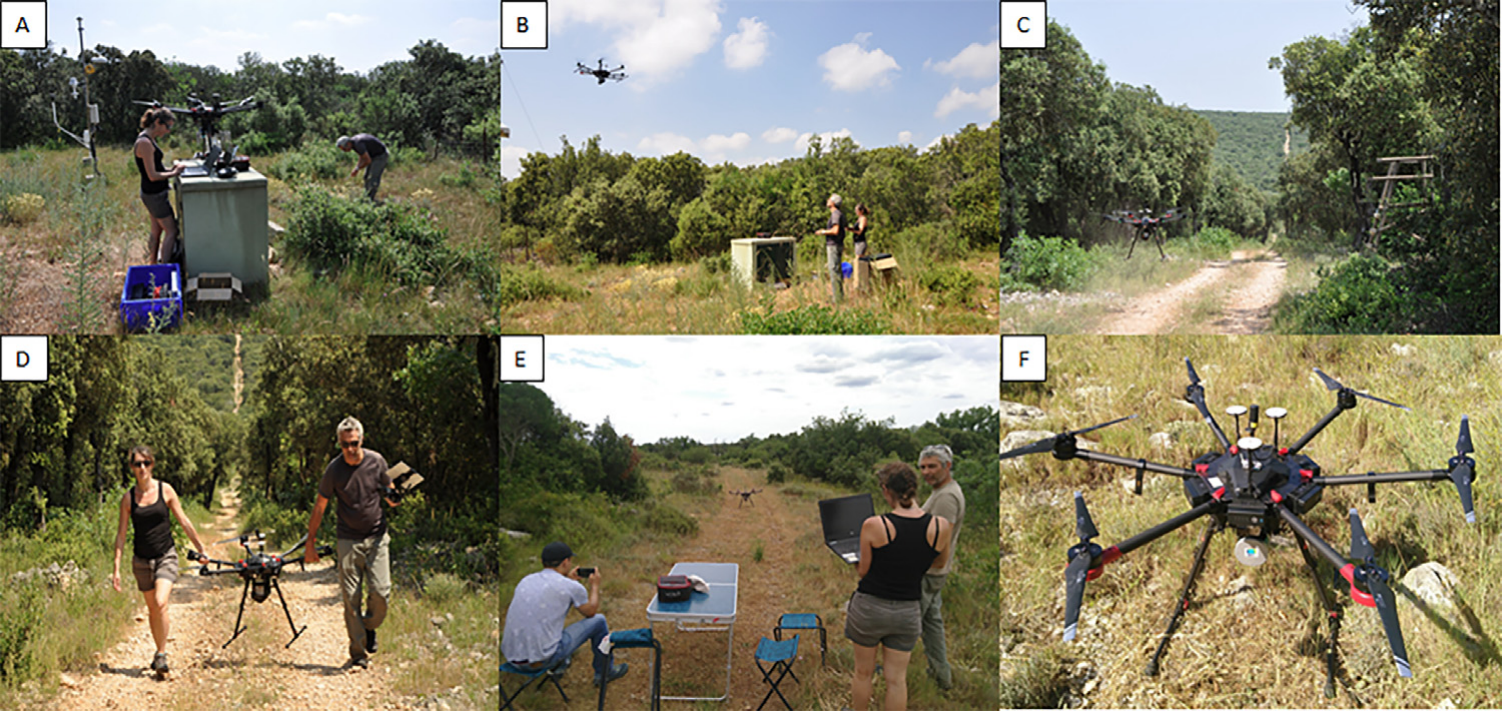
Photos A, B, C and D from the LiDAR overflight campaign at PUE (photos by Damien Longepierre, IRD) and the same for photos E and F at PSL (photos by Josselin Giffard Carlet, INRAE).

For PUE, six flights were carried out on a single day in June 16th, 2021 while for PSL, four flights were carried out on June 23rd and 26th, 2021. More details about the flight configurations are provided in Table 6 and Fig. 20 for both sites. An illustration of the PUE point cloud is given in Fig. 21 and photos of the overflight campaign in Fig. 22.

### 4.3 Airborne and satellite imagery

AVIRIS-Next Generation airborne imagery were acquired on the 9th and 10th of June (cf. Table 4). The first day, the acquisitions were performed over PSL then PUE with spatial resolutions varying between 3.0 m and 3.1 m, and time ranging between 7h59 - 8h19 and 8h23 - 8h31 (UTC) respectively. Thereafter, PSL was overflew at higher spatial resolutions ranging between 1.2 and 1.4 m between 8 h38 and 9 h02. The mission was then aborted due to the presence of incoming clouds. The second day was dedicated to the flights over PUE between 8 h58 and 9 h19 with the same range of high resolutions as the previous PSL acquisitions.

**Fig. 23:**
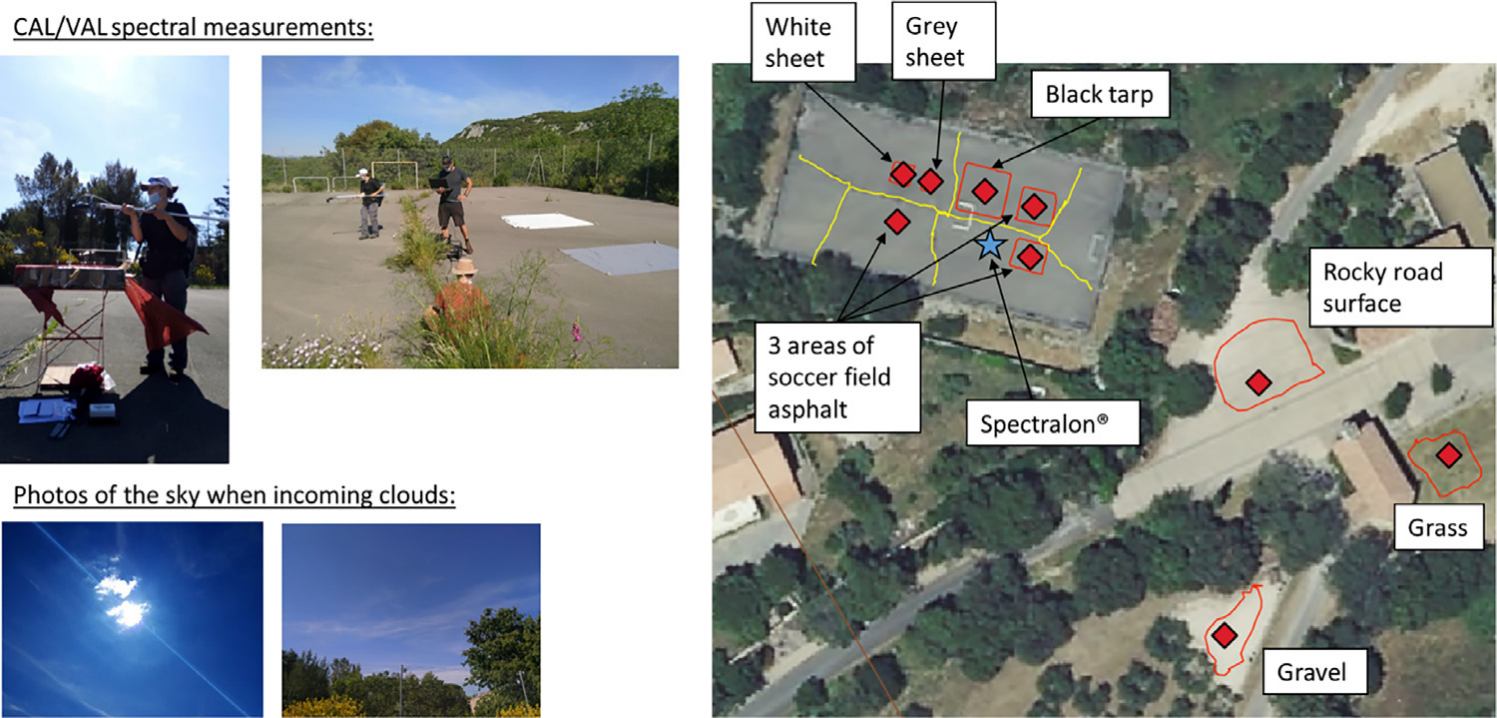
Calibration/validation activities for seven material targets during the AVIRIS-Next Generation flights in downtown Cazevieille city on 9th of June 2021.

On the 9th of June, calibration/validation activities were performed by the MEDOAK crew and was composed of *in situ* spectral measurements with a ASD FieldSpec 3 spectroradiometer and a fore optic of 8° by considering a total of seven targets (Fig. 23):

• Three reference materials (black tarp, light grey sheet and white sheet).
• Two natural surfaces (fine gravel in a park, grass in an open area).
• Two manmade surfaces (asphalt in a soccer field, rocky road surface).

Several reference spectral measurements were taken for calibration with a Spectralon® of known reflectance. These measurements were repeated continuously during the AVIRIS Next-Generation flights. They followed ESA and NASA SGCP field campaign – Design & Preparation, Handling & Operation, Protocol v.2.1. Photos of each material, the surroundings and sky views were taken, as well as GPS acquisitions of the location of these materials. The sky condition were variable, from clear sky to the apparition of cirrus clouds on the horizon, then occasional clouds in front of the sun. These data were used for in-flight calibration correction and the production of L2 at-surface reflectance image products by NASA JPL.

The list of available satellite data for the year 2021 comprises (cf. Table 4):

• 7 PRISMA images, all acquired around 10 h50, four of them within ten days of the field samplings.
• 4 DESIS images, acquired between 8 h11 and 17h01, two of them within three days of the field samplings.
• 48 Sentinel-2 images, all acquired at 10 h49, eight of them within three days of the field samplings.

Airborne and satellite data are not provided but can be directly downloaded on the websites previously quoted in the Specification Table. The different spatial and spectral characteristics are given in Table 7. Their temporal availability in relation to our study sites and the period of study was already mentioned in Table 4.

**Table 7:** General overview of airborne and satellite instrument characteristics in the optical range 400–2500 nm.

Platform	Airborne	Spaceborne		
Instrument	Aviris-Next Generation	Sentinel-2A/2B	PRISMA	DESIS
Acquisition height	Around 1.2 km and depending on spatial resolution	786 km	614 km	400 km
Spatial resolution	1.2–1.4 m 3.0–3.1 m	10 m, 20 m and 60 m	30 m	30 m
Spectral characteristics	Range: 380–2510 nm Sampling: 5 nm Resolution: 5 nm Number of bands: 425	Range: 440–2200 nm Resolution: 15–185 nm Number of bands: 13	Range: 400–2505 nm Resolution: ≤ 12 nm Number of bands: 239	Range: 402–1000 nm Sampling: 2.55 nm Resolution: 3.5 nm Number of bands: 235

## CRediT authorship contribution statement

**K. Adeline:** Conceptualization, Methodology, Validation, Formal analysis, Investigation, Resources, Data curation, Writing – original draft, Supervision, Project administration, Funding acquisition. **J.B. Féret:** Conceptualization, Methodology, Validation, Formal analysis, Investigation, Resources, Data curation, Writing – review & editing, Supervision. **H. Clenet:** Conceptualization, Methodology, Formal analysis, Investigation, Resources, Data curation, Writing – review & editing, Supervision. **J.M. Limousin:** Conceptualization, Methodology, Formal analysis, Investigation, Resources, Data curation, Writing – review & editing, Supervision. **J.M. Ourcival:** Conceptualization, Methodology, Formal analysis, Investigation, Resources, Writing – review & editing. **F. Mouillot:** Conceptualization, Methodology, Investigation, Writing – review & editing. **S. Alleaume:** Conceptualization, Methodology, Validation, Formal analysis, Investigation, Resources, Data curation, Writing – review & editing, Visualization. **A. Jolivot:** Methodology, Formal analysis, Investigation, Resources, Writing – review & editing. **X. Briottet:** Conceptualization, Methodology, Validation, Formal analysis, Investigation, Writing – review & editing. **L. Bidel:** Methodology, Formal analysis, Investigation. **E. Aria:** Investigation. **ATM. Defossez:** Investigation. **T. Gaubert:** Investigation. **J. Giffard-Carlet:** Validation, Formal analysis, Investigation, Data curation. **J. Kempf:** Investigation. **D. Longepierre:** Validation, Investigation, Data curation, Writing – review & editing. **F. Lopez:** Investigation. **T. Miraglio:** Investigation. **J. Vigouroux:** Investigation. **M. Debue:** Validation, Formal analysis, Writing – review & editing, Visualization.

## Data Availability

SENTHYMED/MEDOAK datasets (Original data) (SEDOO).
